# Diverse nitrogen acquisition strategies of conifer-associated ectomycorrhizal fungi shape unique responses to changing nitrogen regimes

**DOI:** 10.3389/fpls.2025.1666003

**Published:** 2025-09-23

**Authors:** Lotus Lofgren, François Maillard, Talia Michaud, Alice Gredeby, Anders Tunlid, Peter G. Kennedy

**Affiliations:** ^1^ Department of Plant and Microbial Biology, University of California, Berkeley, Berkeley, CA, United States; ^2^ Microbial Ecology, Department of Biology, Lund University, Ecology Building, Lund, Sweden; ^3^ Department of Plant and Microbial Biology, University of Minnesota, Saint Paul, MN, United States

**Keywords:** suillus, nitrogen, genomics, isotopes, organic, inorganic, chitin, protein

## Abstract

Ectomycorrhizal fungi are critical mediators of nitrogen acquisition in forest ecosystems, exhibiting variation in both host association and metabolic traits that mediate differential responses to forest nitrogen availability. However, how nitrogen acquisition strategies vary among closely related fungal species, how these patterns manifest in conifer-associated ECM fungi, and whether they persist over changing nitrogen regimes, remains poorly understood. Using an integrative approach combining *in silico* genomic analysis, *in vitro* growth assays, and isotopic analysis of *in situ* specimens spanning six decades, we provide the first comprehensive examination of nitrogen assimilation in congeneric conifer-associated ectomycorrhizal fungi using six *Suillus* species. We found highly conserved genes for inorganic nitrogen assimilation across species, but striking interspecific variation in the genetic capacity for organic nitrogen metabolism. Interspecific differences were also observed in fungal growth on varying nitrogen substrates in the growth assays, as well as in the isotopic signatures of historical specimens. For the latter, carbon isotopic patterns showed divergent temporal trends among *Suillus* species, suggestive of differential N use over time. Collectively, these genomic, physiological, and isotopic findings support the presence of notable interspecific diversity in ectomycorrhizal fungal nitrogen acquisition and suggest that coniferous forests and their fungal symbionts exhibit distinct responses to shifts in nitrogen availability compared to broadleaf forests. The ability of even closely related ectomycorrhizal fungi to employ diverse nitrogen acquisition strategies has important implications for forest ecosystem resilience, as different species may provide complementary services to host trees under varying environmental conditions, potentially reducing competition, and influencing forest responses to altered nutrient availability.

## Introduction

Nitrogen (N) is an essential nutrient for plant growth, but its availability in terrestrial ecosystems is often limited. Despite being highly abundant in gaseous form, only a small proportion of the total N pool exists in inorganic forms such as ammonium and nitrate, which are the forms most readily available to plants (Tamm, 1991). Under these nutrient-constrained conditions, many trees have evolved symbiotic associations with ectomycorrhizal (ECM) fungi, which trade N and other critical nutrients in exchange for carbon (C) fixed by the host during photosynthesis (Smith and Read, 2010). These fungal symbionts substantially enhance plant N acquisition both by expanding the surface area and soil volume explored, and by metabolizing organic sources of soil N such as most amino acids, peptides, protein, and chitin that are not are not directly accessible to plant hosts (Chalot and Brun, 1998; Nicolás et al., 2019; Maillard et al., 2023).

While these symbioses represent critical adaptive strategies for plant nutrient acquisition, the environmental conditions under which these strategies evolved are changing. Recent evidence suggests that terrestrial nutrient availability has been declining for decades, a process termed terrestrial oligotrophication (McLauchlan et al., 2010, 2017; Craine et al., 2018; Mason et al., 2022). These declines are attributed to increasing atmospheric CO_2_, which enhances primary production via increased photosynthetic rates and extended growing seasons (Groffman et al., 2018). Although atmospheric N deposition could theoretically mitigate declining terrestrial N status, empirical evidence indicates continued oligotrophication despite increased N inputs. This paradox is thought to be driven by several interacting factors including how different N sources interact with microbial communities. While the predominant N source in forest soils is organic (representing up to 95% of the total N pool) (Pena and Tibbett, 2024), atmospheric N deposition predominantly occurs in inorganic forms, thus altering the ratios of inorganic:organic N accessible to ECM host plants and their fungal symbionts. Many ECM fungi are sensitive to fluctuations in N availability, with increases in the inorganic N fraction associated with altered fungal community composition, reduced fungal diversity, decreases in both ECM fungal colonization of host root tips and the production of sporocarps, and shifts in facultative interactions along the mutualism-parasitism spectrum (Arnolds, 1991; Hasselquist and Högberg, 2014; Wang et al., 2022; Peng et al., 2022; Tang et al., 2024).

Despite the recognized importance of ECM fungi in N cycling, major gaps remain in our understanding of their functional diversity (Koide et al., 2007). In particular, the extent to which genomic mechanisms of N assimilation are conserved across fungal taxa, and how this variation scales to ecosystem-level N cycling, remains largely unresolved. Understanding interspecific differences in N metabolic strategies is essential for predicting ecosystem resilience under environmental change, yet it remains unclear whether ECM fungi exhibit interspecific variation in N acquisition strategies and how genetic divergence translates to phenotypic plasticity under changing N regimes.

These dynamics are particularly consequential in coniferous forests, where both hosts and their ECM fungal symbionts are thought to be highly N-sensitive (van der Linde et al., 2018; Lilleskov et al., 2019), suggesting potentially divergent responses to atmospheric N inputs compared to broadleaf forests. While research has identified century-scale signatures of declining N and δ^15^N isotopes among both broadleaf host trees and their associated ECM fungi despite moderate levels of N deposition (Michaud et al., 2024), the response of coniferous species remains unexplored. Further, because different ECM fungi associate with different hosts and exhibit distinct functional traits, shifts in forest community composition can interact bidirectionally with biogeochemical processes. In particular, high levels of inorganic N select against N sensitive taxa which may be more adept at metabolizing organic N sources, but outcompeted by fungi that can take advantage of increased levels of inorganic N. The functional traits demonstrated by the species that remain can result in decreased fungal biomass inputs, altered enzymatic potential, and changes to rates of organic matter decomposition (Wang et al., 2022; Gao et al., 2024; Jörgensen et al., 2025).

The genus *Suillus*, a widespread conifer-associated genus of ECM fungi, has long served as a model for studies of fungal ecology and evolution (Lofgren et al., 2024). As such, historical collections of *Suillus* sporocarps (mushrooms) are well represented in fungal herbaria, providing opportunities to evaluate N use and N availability over time. Elemental analysis of sporocarp tissue has been used to evaluate fungal nutritional status, N availability, and trophic state (Hasselquist and Högberg, 2014; Kranabetter et al., 2019; Hobbie et al., 2019). Specifically, enrichment of ^15^N is thought to be associated with the utilization of organic N, and N sourced from deeper and older soil profiles, while ^13^C patterns in fungal tissues can also reflect the incorporation of C from organic N compounds versus recent photosynthates (Hobbie and Ouimette, 2009; Hobbie and Högberg, 2012; Michaud et al., 2024). Additionally, due to differences in protein concentration and isotopic fractionation, cap (pileus) tissue generally contains lower isotopic values than stipe tissue (Hobbie et al., 2012). This phenomenon can be used to help distinguish the source of ^15^N enrichment, contrasting internal processes like the preferential allocation of N to proteins vs chitin, and external processes like the metabolism of N sources that naturally contain more ^15^N. The combination of *Suillus*’ ecological importance and high host-specificity with plants in the family Pinaceae, the availability of high-quality genome assemblies, tractability to laboratory growth assays, and extensive herbarium collections, makes *Suillus* an ideal model for investigating conifer-associated ECM fungi in the context of changing N regimes.

Characterizing the complex interactions among plants, fungi, and edaphic nutrient availability is crucial for understanding N cycling in terrestrial ecosystems and predicting the consequences of long-term environmental change. Here, we assessed the capacity for inorganic and organic N assimilation across six conifer-associated ECM *Suillus* fungi using a combination of genomics, growth assays on different N sources, and elemental analysis of historical host tree and fungal collections. Specifically, we addressed three key questions: 1) How conserved are key genetic modules for inorganic versus organic N assimilation across closely related ECM fungi? 2) Do interspecific genomic differences correspond with functional performance under controlled conditions? 3) Do conifer-associated ECM fungi and their hosts exhibit expected temporal patterns of declining N status over time, or do they respond differently to changing N availability? Our results revealed highly conserved genes for inorganic N acquisition but striking interspecific variation in genes for organic N acquisition. This genomic variability corresponded with differential growth performance when cultured on various N sources. Additionally, the isotopic signatures from our historical collections confirmed these interspecific differences and supported the conclusion that coniferous forests and their associated ECM symbionts likely respond differently to altered N availability compared to broadleaf forests.

## Methods

### Strain selection

We selected six species of *Suillus* based on their occurrence in forests in Minnesota, USA, the availability of cultures for genome-sequenced strains, and the availability of historical herbarium data for both the fungi and their associated hosts. These species included *S. weaverae* EM37, *S.* sp*raguei* EM44, *S. americanus* EM31, *S. clintonianus* FC179, *S. ampliporus* FC55, and *S. luteus* UH-Slu-Lm8-n1. All genomes were originally sequenced as part of Lofgren et al., 2021 (Lofgren et al., 2021), except for *S. luteus* UH-Slu-Lm8-n1, which was sequenced as part of Kohler et al. (2015). Whereas, *S. americanus*, *S. weaverae*, and *S.* sp*raguei* specialize on white pine (*Pinus* sg. Strobus), *S. luteus* associates with red pine (*Pinus* sg. Pinus), and *S. clintonianus* and *S. ampliporus* associate with tamarack (*Larix*) (Nguyen et al., 2016).

### Phylogenetic assessment

The phylogenetic relationship between the six *Suillus* species was estimated using PHYling v.2 (Stajich and Tsai, 2023), employing the BUSCO conserved single-copy marker set (fungi_odb10). Reconstruction was accomplished using IQTree v.1.6.12, with model selection using ModelFinder (Kalyaanamoorthy et al., 2017). The optimal model based on BIC score was VT+F+R4, which was run with 1000 replicates of Ultrafast Bootstrapping, on a total of 35,550 parsimony-informative sites. AMT gene-tree reconstruction was accomplished by aligning all AMT genes using MAFFT v.7 (Katoh and Standley, 2013), with subsequent alignment trimming using ClipKIT (Steenwyk et al., 2020), removing a total of 34 sites. The best model for AMT gene reconstruction was determined to be JTT+G4 according to ModelFinder, and IQTree was run on a total of 312 parsimony informative sites.

### *In silico* genomic analyses

To investigate the genomic capacity of *Suillus* species to use inorganic N sources, we conducted genome mining and comparative genomic analysis for genes involved in the metabolism of both ammonium and nitrate. Genes involved in ammonium assimilation were identified via homology to the three characterized ammonium transporter (AMT) genes in *Hebeloma cylindrosporum* (Javelle et al., 2001, 2003) HcAmt1 (GenBank ID AAM21926), HcAmt2 (AAK82416), HcAmt3 (AAK82417). BLASTP searches were conducted as above, first to identify the relevant orthogroups, and then to assign and quality filter individual orthogroup members. After one orthogroup was found to be conspicuously missing a gene from *S. ampliporus*, an additional whole-proteome search was conducted to determine the loss status of this gene in the *S. ampliporus* assembly, with BLASTP search parameters set as above.

Genes involved in nitrate assimilation were identified via homology to characterized genes in *Laccaria bicolor*, including those encoding nitrate transporters (*Lbnrt*, protein ID 254042), nitrate reductase (*Lbnr*, ID 254066), and nitrite reductase (*Lbnir*, ID 291348) (Kemppainen et al., 2010). Initial BLASTP searches were run as above using representative proteins for every orthogroup to identify the above gene families, and then run on all sequences contained in those orthogroups to implement stringent quality filtering using the cutoffs for identifying putative proteases in the MEROPS database (see below). Sequences of each quality filtered orthogroup along with their corresponding *L. bicolor* reference genes and exons were aligned using MAFFT v.7 (Katoh and Standley, 2013) with the parameters –maxiterate 1000, –localpair, and analyzed using Jalview (Waterhouse et al., 2009).

To investigate the genomic capacity of *Suillus* species to utilize organic N sources, we conducted genome mining and comparative genomic analysis of genes involved in both and protein and chitin catabolism. Identification of genes involved in protein catabolism was accomplished using orthology to protease type examples housed in the MEROPS database (Rawlings et al., 2018). First, we used OrthoFinder to cluster gene families (Emms and Kelly, 2019), retaining the longest sequence in each family as a representative for each orthogroup. We then removed annotated inhibitors from the MEROPS SCAN library v.12.5 (n = 673 inhibitors) and used the remaining .fasta files (n = 4,335) to create a curated database of non-redundant proteases. We used BLASTP to search for matches between the sequence representatives for each orthogroup and the protease database with parameters e-value = 1e-10 and max_target_seqs = 10. We implemented subsequent QC filtering to retain only results with >40% sequence identity, >70% query coverage, >70% target coverage, and retained only the top hit according to e-value in the case of multiple matches. Annotations were applied to these results by mapping the protein target IDs to MEROPS database annotations.

For the chitin assessment, we annotated CAZymes and Auxiliary Activity (AA) enzymes based on orthology to all currently accepted CazyDB CAZyme classes accessed via the MycoCosm web portal (Levasseur et al., 2013; Grigoriev et al., 2014; Drula et al., 2022). Recent advances have revealed that chitin degradation involves enzymes beyond classically studied families, with members such as GH2, GH9, GH16, GH23, and GH35 demonstrating activity on chitin-derived substrates (Viborg et al., 2019; Vaaje-Kolstad et al., 2019; Zeng et al., 2025) Identification of relevant CAZyme families was accomplished via the manual curation of CazyDB and CAZypedia annotations of all functionally characterized enzymes and associated carbohydrate-binding modules active on chitin, chitosan, and chitin degradation derivatives (CAZypedia Consortium, 2018; Drula et al., 2022). This comprehensive approach captures chitin-active enzymes beyond canonical chitinases and ensures we do not overlook potentially significant enzymatic activities contributing to fungal chitin metabolism. These included the endo-acting enzymes Chitinase (EC 3.2.1.14), Chitin deacetylase (EC 3.5.1.41), Chitin oligosaccharide deacetylase (EC 3.1.1.-), N-acetylglucosamine-6-phosphate deacetylase (EC 3.5.1.25), and the chitin active lytic polysaccharide monooxygenases (LPMOs) encoding Lytic chitin monooxygenase (EC 1.14.99.53). To assess exo-acting enzymes relevant to chitin metabolism, we included Chitin exo-β-1,4-N-acetylglucosaminidase (EC 3.2.1.-), Chitin exo-β-1,4-N-acetylglucobiosaminidase (EC 3.2.1.200), Di-N-acetylchitobiase/reducing-end exo-hexosaminidase (EC 3.2.1.-), [reducing end] exo-chitinase (EC 3.2.1.201), β-1,6-N-acetylglucosaminidase (EC 3.2.1.-), Chitin β-1,3/1,6-glucanosyltransferase (EC 2.4.1.-) and β-N-acetylhexosaminidase (EC 3.2.1.52). To assess chitosan metabolism we included Chitosanase (EC 3.2.1.132), and Exo-β-1,4-glucosaminidase (EC 3.2.1.165).

### *In vitro* growth assays

To evaluate how well our genome-based predictions of N use matched metabolic activity *in vitro*, we measured biomass production of the same six genome-sequenced *Suillus* speices when grown on diverse N sources. These sources included inorganic N in the form of ammonium, and organic N in the form of protein, protein-tannin complexes, protein-mineral complexes, and chitin. Cultures of each species were first grown for six weeks on a sterile cellophane membrane covering solid modified Fries media at 21 °C in the dark. The modified Fries media contained 2.72 mM (NH_4_)2•tartrate, 2.05 mM MgSO_4_·7H_2_O, 5.88 mM KH_2_PO_4_, 0.18 mM CaCl_2_·2H_2_O, 0.34 mM NaCl, 1, 0.24 mM H_3_BO_3_, 20 μM ZnSO_4_·7H_2_O, 5.01 μM CuSO_4_·5H_2_O, 50.29 μM MnSO_4_·H2O, 0.16 μM (NH_4_)_6_Mo_7_O_24_·4H_2_O, 73.99 μM FeCl_3_·6H_2_O, 55.5 mM d-glucose, 55.51 μM myo-inositol, 0.30 μM thiamine·HCl, 0.10 μM biotin, 0.59 μM pyridoxine·HCl, 0.27 μM riboflavin, 0.82 μM nicotinamide, 0.73 μM p-aminobenzoic acid, and 0.46 μM Ca-pantothenate, supplemented with 0.23 g/L of casein enzymatic hydrolysate and 0.5 g/L of yeast extract (Fries et al., 1987; Op De Beeck et al., 2018). The pH of the medium was adjusted to 4.8 using HCl, and agar was added at a concentration of 12 g/L.

To conduct the N use assay, a single layer of autoclaved glass beads was added into 6-well plates as described in Maillard et al (Maillard et al., 2023). Modified Fries medium (made as above) was used as the base medium, omitting the casein enzymatic hydrolysate and the yeast extract, and with a lower (NH_4_)2•tartrate concentration of 0.109 mM. A treatment using the base medium without any additional N supplementation served as a control to account for fungal growth using solely (NH_4_)2•tartrate and any N potentially contained in the vitamin and micro-nutrient solutions. The other treatments included the base medium supplemented with (NH_4_)2•tartrate (0.460 g/L), bovine serum albumin (BSA) (0.437 g/L), and chitin from shrimp shells (1 g/L). (NH_4_)2•tartrate was used as an inorganic N source (Chalot and Brun, 1998). BSA was used as a model for protein degradation, having been previously used to demonstrate organic N acquisition from proteins by ECM fungi (Shah et al., 2013; Wang et al., 2020, 2021; Op De Beeck et al., 2018) and chitin from shrimp shells was used as in Maillard et al (Maillard et al., 2018, 2023) to study the degradation of chitin as a source of organic N. In forest soils, proteins occur not only freely but can also form complexes with phenolic molecules or absorb onto mineral surfaces (Adamczyk et al., 2017), which limits their biodegradability (Knicker et al., 1999; Jilling et al., 2018). Thus, to represent the complexity of protein forms in soils, we also included protein-tannin and protein-mineral complexes as organic N sources. Protein-tannin complexes were prepared as described in Bending and Read (1996), and protein-mineral complexes were prepared as described in Wang et al. (2021) using goethite, a naturally abundant iron oxide in soils as a model mineral. For the protein-tannin and protein-mineral treatments, the BSA concentration was the same as in the free-BSA treatment (0.437 g/L), with almost all BSA considered complexed with tannins or absorbed on geothire based on Bending and Read (1996) and Wang et al. (2021). The respective concentrations of (NH_4_)2•tartrate, BSA (either free, complexed, or absorbed), and chitin were chosen to contain the same mass of N per treatment, and to be N-limited with a C:N ratio of approximately 28.6, making the additional C incorporated in chitin, protein, tartrate, or tannic acid negligible relative to glucose C. We added 2.4 mL of each of the five media treatments (low NH_4_ control, NH_4_, free-BSA, BSA-tannin, and chitin) into the glass-bead-covered wells of 6-well plates. For each of the *Suillus* species, we cut small mycelium disks (3 mm in diameter) from the cellophane membrane close to the hyphal front and placed one disk in each well (n = 3 wells per treatment). The plates were incubated in the dark at 21 °C and harvested at three timepoints (12-, 24-, and 35-days post-inoculation). The mycelium collected was quickly blotted on a paper towel, then dried at 50 °C, and the dry biomass measured on an analytical balance. To control for variation in the amount of starting material, the dry mass of the initial transplant (day 0) was estimated by averaging 10 transplants per species. To account for any free goethite in the BSA-goethite treatment, all *Suillus* biomass grown with BSA-goethite as an N-source were combusted at 550 °C for 12 hours in a furnace. Goethite alone was combusted to account for goethite mass loss during combustion. Samples from the ammonium treatment (high biomass) were also combusted to evaluate fungal biomass remaining after 12 hours in 550 °C, and determined to be negligible. The goethite contribution in the original fungal biomass samples was then subtracted to correct the fungal biomass values. Three values were considered outliers (all more than 5 times the average) and subsequently excluded (n=1 *S. clintonianus*, n=1 *S.* sp*raguei* and n=1 *S. weaverae* all at the 35 day time point).

Differences in fungal biomass production across treatments and species were analyzed using a two-way ANOVA with Type III sum of squares, implemented in R (version 4.4.1). To assess relative biomass production across treatments, we first normalized the measurements by subtracting the dry weight of the initial transplant. Then, for each species at each time point, we averaged the biomass generated on the low ammonium control media across n=3 replicates, and subtracted it from each of the treatment biomass measurements for the corresponding time point. Normal distribution of residuals and homogeneity of variance were confirmed using visual inspection. Effect sizes were calculated as eta-squared values (proportion of total variance explained by each factor) by dividing the sum of squares for each factor by the total sum of squares. *Post-hoc* comparisons were conducted using Tukey’s HSD to identify significant differences between species’ performances within each treatment and between treatments for each species.

### *In situ* elemental and isotopic analyses

In addition to ^15^N, the analysis of ^13^C isotopes can provide indirect evidence of N source utilization. The progressive depletion of atmospheric ^13^C compared to ^12^C, due to the release of anthropogenic emissions into the atmosphere is known as the Suess effect (Bassett et al., 2023). These ratios are reflected in plants and other carbon reservoirs, including sporocarps (mushrooms). Historically, plant δ^13^C declined slowly from 1850–1957 during the early industrial revolution, and began declining rapidly starting in 1957 coinciding with globalization, industrialization, and massive increases in fossil fuel consumption (Belmecheri and Lavergne, 2020). By comparing δ^13^C composition of the same species across time, we can also infer N use, where more negative δ^13^C values indicate the assimilation of older, organic, forms of N. We therefore considered both δ^13^C and δ^15^N in our isotopic evaluation.

A total of 112 *Suillus* mushrooms and 131 host plant specimens were selected from the collections at the Bell Herbarium at the University of Minnesota (**Supplementary Table S1**). All specimens were collected in Minnesota between 1961-2021. Fungal caps and stipes were sampled and processed separately. To conduct elemental and isotopic analysis, air-dried sporocarp and needle tissues were ground and a mass of 2 ± 0.05 mg to the nearest 0.001 mg was analyzed via an Elementar Cube elemental analyzer interfaced to a GV Instruments Isoprime isotope ratio mass spectrometer (Manchester, UK) at Boston University Stable Isotope Laboratory.

Due to normal isotopic fractionation during N metabolism and transport, cap tissue should exhibit lower isotopic values than stipe tissue (Hobbie et al., 2012). As such, samples where δ^15^N or δ^13^C were lower in stipe than cap were considered aberrant, potentially indicating contamination, abnormal physiological processes, or sample preservation issues. Sporocarps failing this quality control criteria were therefore excluded from our analysis, resulting in the removal of 5 samples (n=1 *S. americanus*, and n=2 of each *S. clintonianus* and *S. weaverae*). After removing these samples, the final dataset included n = 20 samples of *S. americanus*, n = 11 of *S. ampliporus*, n = 24 of *S. clintonianus*, n = 9 of *S. luteus*, n = 15 of *S.* sp*raguei*, and n = 28 of *S. weaverae*. The host dataset consisted of n = 55 samples of *L. laricina*, n = 34 of *P. resinosa*, and n = 42 P*. strobus*.

For all downstream modeling, values obtained for caps and stipes were averaged for each sporocarp. Mixed effects models were constructed in R to model sporocarp and needle N concentrations, δ^15^N, and δ^13^C for both plant and fungal tissues. For all *Suillus*, to account to non-independence arising from location and seasonality, county and month collected in were included as random effects. Year was included as a fixed effect in all models to test for temporal trends, as well as an interaction between year and species, and species as a fixed effect to account for species-level variation in baseline values. Backwards AIC stepwise selection was performed on all models. Month information was missing for four *Suillus* collections, which were removed prior to stepwise selection, and reincluded if month was eliminated as a random effect. In many models, stepwise selection eliminated county and month as random effects. These models were then run using simple linear regression via the “lm” function in base R with the preserved variables. To model the δ^15^N difference between cap and stipe, sporocarp average N concentration and the difference between cap and stipe N concentrations were included as fixed effects to test the effect of overall N status, and the amount of protein in the cap, respectively.

## Results

### Phylogenetic assessment

**Figure 1:**
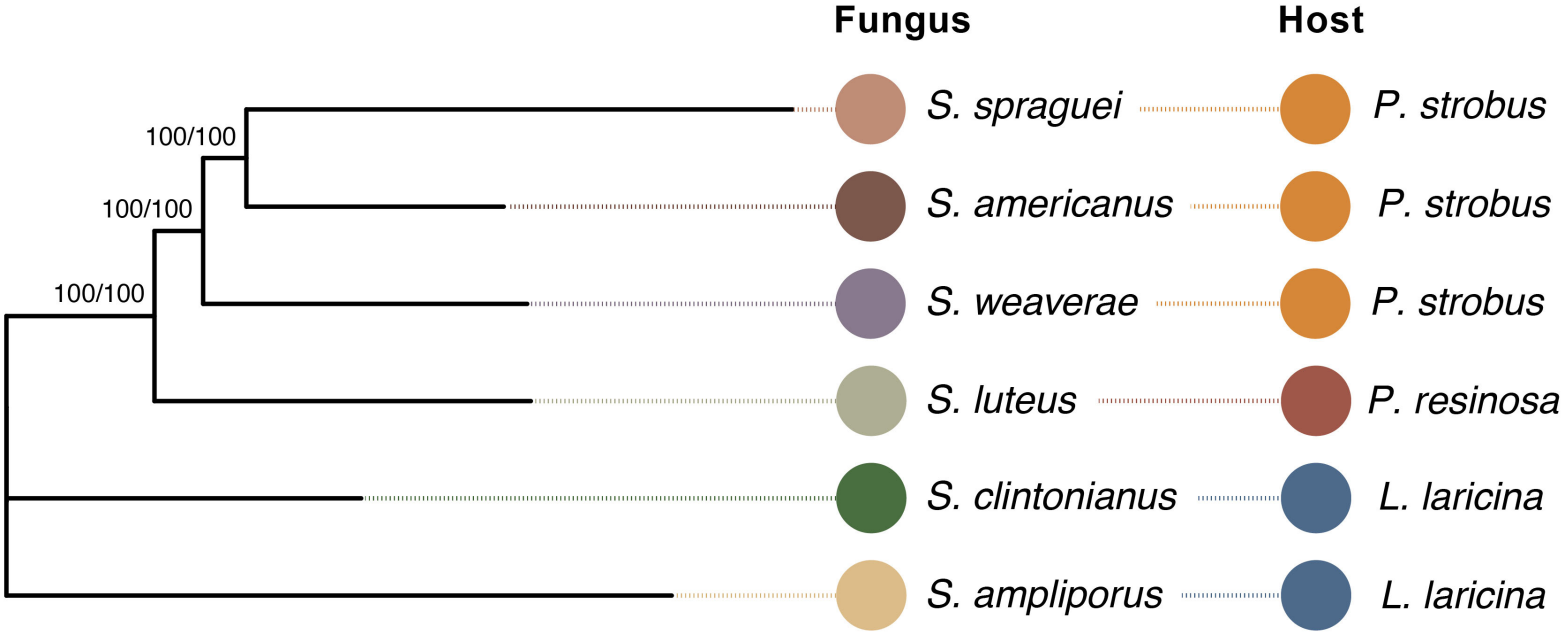
*Phylogenetic reconstruction of six* Suillus sp*ecies.* Tree building was accomplished using PHYling v2, employing the BUSCO conserved single-copy marker set and IQTree v.1.6.12 with model VT+F+R4, run across 35,550 parsimony-informative sites. Branch support values (UFBoot/SH-aLRT, 1000 iterations each) are shown at each split, except where rooted at the node leading to *S. ampliporus.* Colored circles represent fungal species (left) and their corresponding host species (right): *S.* sp*raguei*, *S. americanus*, and *S. weaverae* associate with eastern white pine (*Pinus strobus*), *S. luteus* with red pine (*P. resinosa*), while *S. clintonianus* and *S. ampliporus* associate with larch (*Larix laricina*). Fungal and host species designations are highlighted by the same color scheme throughout the study.

Phylogenetic analysis of the six *Suillus* species revealed well-supported evolutionary relationships with all nodes receiving maximum bootstrap support (**Figure 1**). *S.* sp*raguei*, *S. americanus*, and *S. weaverae* form a strongly supported monophyletic group, while *S. luteus* occupies a sister position to this trio. In agreement with previous work, all species exhibit distinct evolutionary lineages that correspond to their documented host associations (Nguyen et al., 2016; Lofgren et al., 2021).

### *In silico* genomic analysis

#### Inorganic N

**Figure 2:**
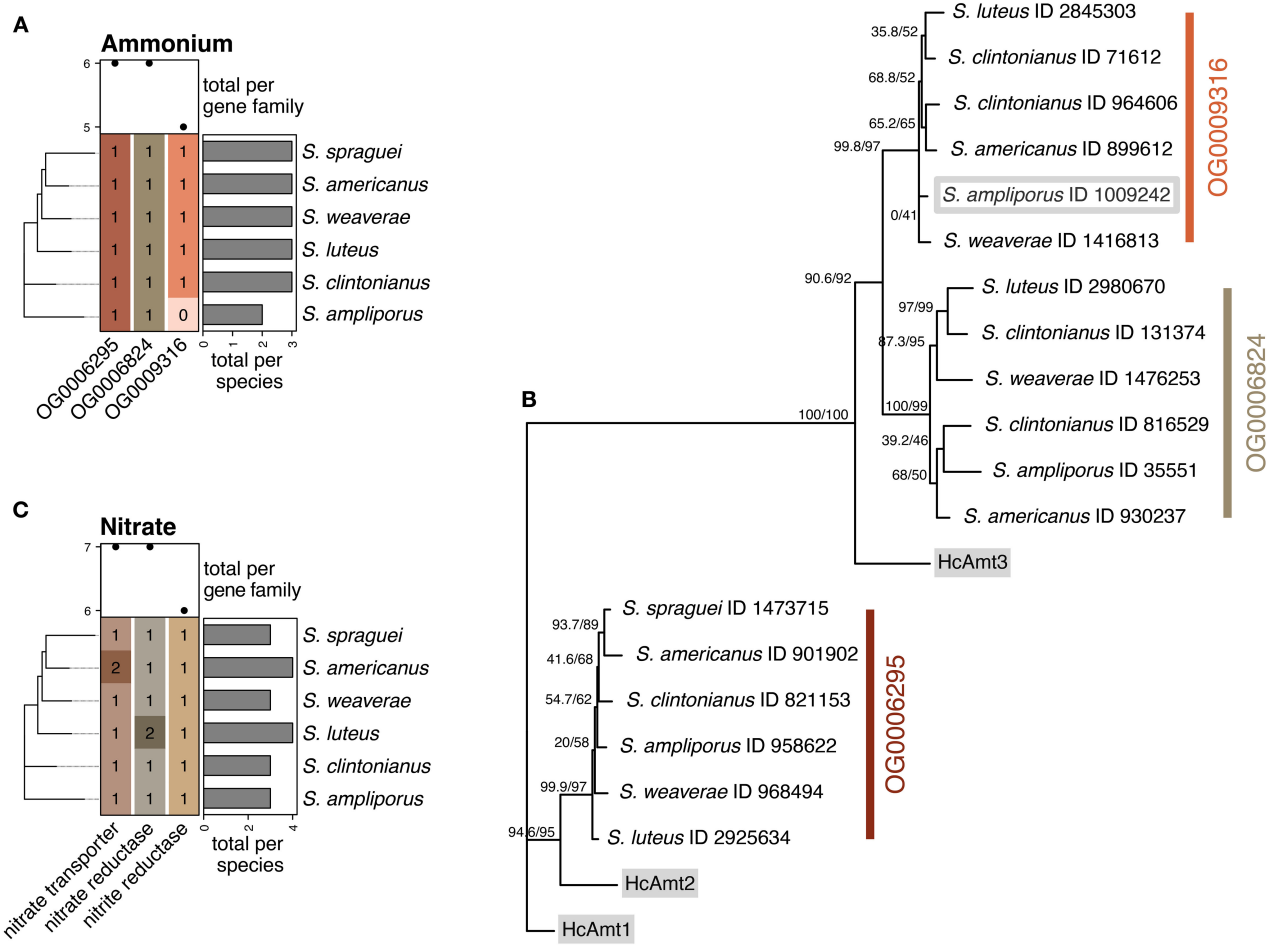
*Copy number variation and distribution of genes involved in inorganic nitrogen acquisition.* **(A)** The presence of genes involved in ammonium assimilation was assessed by looking at orthology to the characterized ammonium importers AMT1, AMT2, and AMT3. Three orthogroups were identified containing high identity matches to the AMT superfamily using BLASTP, but could not be confidently assigned to their corresponding high- and low-affinity AMT importers. These genes were single copy in all 6 species, except for one gene in *S. ampliporus* which was missing from OG0009316. Further analysis identified a truncated and putatively non-functional gene model in *S. ampliporus* with a significant BLASTP match to the AMT superfamily, and phylogenetic reconstruction **(B)** of AMT genes along with reference HcAmt genes from *H*. *cylindrosporm* (grey highlights), clustered this gene with the other single-copy genes in orthogroup OG0009316 (grey outline). Reconstruction similarly identified three groups of AMT genes in *Suillus*, but was unable to confidently assign these clusters to AMT1, AMT2 or AMT3 based on similarity to the reference. **(C)** Analysis of nitrate assimilation pathway genes including nitrate transporters, nitrate reductase and nitrite reductase genes. All genes appeared to be single-copy with the exception of a nitrate transporter in *S. americanus* which contained a truncation and lack of start site (likely a pseudogene), and nitrate reductase in *S. luteus*, which contained two putatively functional copies.

Genes for ammonium uptake were present in three separate orthogroups (**Figure 2A**). Initial BLASTP screening using the longest sequence representative for each orthogroup was unable to distinguish between subfamilies with significant matches to all three members of the AMT superfamily (HcAmt1, HcAmt2, and HcAmt3). These three orthogroups contained exactly one significant match for each species of *Suillus*, except for orthogroup OG0009316, which lacked a representative from *S. ampliporus*. A subsequent BLASTP search against the entire *S. ampliporus* proteome identified a single additional gene (ID 1009242) with significant hits to all three HcAmt reference genes, but containing a truncation spanning 327 sites of the trimmed alignment. If the gene model is accurate, ID 1009242 is likely non-functional. While we excluded this gene from the AMT gene counts, we included it in the gene-tree to evaluate phylogenetic placement relative to other orthogroup members. The reconstruction clustered ID 1009242 with sequences from the orthogroup otherwise missing a representative of *S. ampliporus* (**Figure 2B**). Excluding this gene, AMT genes appear to be single-copy in *Suillus*. Single-copy conservation of AMT genes has been previously reported in *H. cylindrosporum* (Javelle et al., 2003). Conversely, the AMT superfamily appears to be expanded in *L. bicolor*, with eight gene models present in the reference Lacbi1 assembly (Lucic et al., 2008). However, the functional impact of this expansion on ammonium assimilation is unknown.

Genes for nitrate transporters, nitrate reductase, and nitrite reductase were found in three separate orthogroups. These genes were present in all species and single-copy except in the case of nitrate transporters in *S. americanus* (which matched both gene ID 103411 and ID 1092257) and nitrate reductase in *S. luteus* (which matched both gene ID 14770 and ID 2924285) (**Figure 2C**). Protein alignments of *Suillus* N transporters to *L. bicolor* exons revealed that *S. americanus* gene ID 103411 contained a truncation spanning a total of 154 amino acids covering the start site, exon 1 and most of exon 2, just missing our QC cut off at 71% subject overage. If the model for gene ID 103411 is correct, it is likely a pseudogene. Conversely, the *S. luteus* nitrate reductase genes were largely intact, with subject coverages of 100 and 99 percent for gene ID 14770 and ID 2924285, respectively. Alignment of nitrate reductase genes showed a variable region directly after the start site in exon 1 of the reference, covering up to 49 amino acids that was present in most *Suillus* species, but not in *S. luteus* ID 2924285. However, given that this region was also absent in the reference *L. bicolor* gene, it unlikely to be necessary for function. Overall, *S. luteus* ID 14770 and ID 2924285 displayed high sequence conservation and a percent identity of 89.8% compared to one another. This is in contrast to a percent identity of 53.2% and 53.1% between *S. luteus* 2924285 and *S. luteus* 14770 and the *L. bicolor* reference, respectively. The two *S. luteus* genes also had the same number of predicted exons (19 in each, compared to only 11 in *L. bicolor*, and 17–20 in other *Suillus* species). Given this, *S. luteus* 14770 and *S. luteus* 2924285 appear to be a product of duplication and divergence with unknown impacts on gene function.

### Organic N

#### Protein

**Figure 3:**
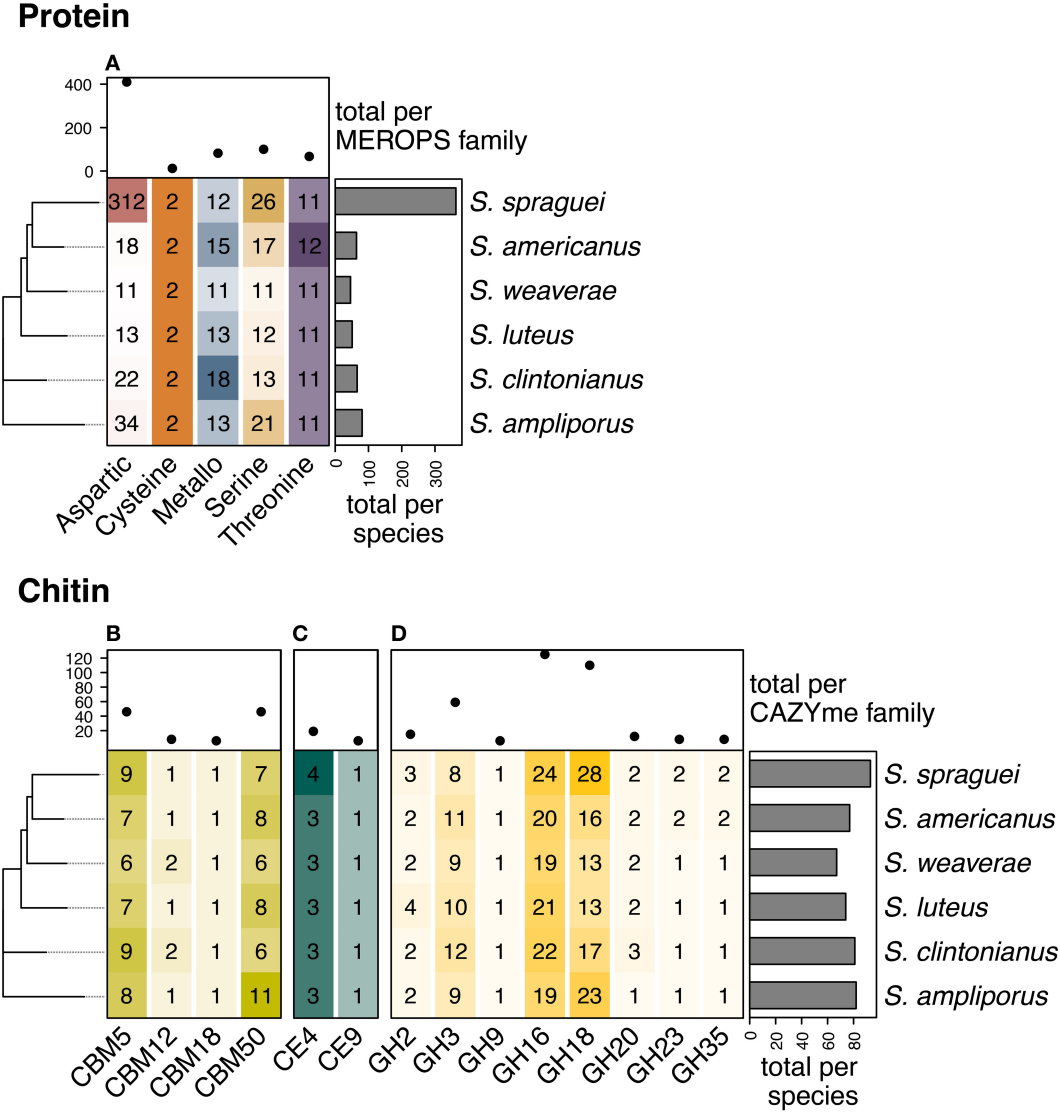
*Copy number variation for genes involved in organic nitrogen acquisition.* Genes involved in the liberation of organic nitrogen (N) and protein **(A)** from chitin **(B-D)**, displayed diversity in both identity and copy number across *Suillus* speciesThe most abundant proteases included those with **(A)** and aspartic (n = 410), serine (n = 100), and metallo (n= 82) key residues, where the abundance of aspartic proteases was largely driven by copy number amplification in *S.* sp*raguei*. We identified 474 genes with roles in the binding and degradation of chitin, chitosan, and associated derivatives including **(B)** carbohydrate-binding modules (CBMs, n = 106), **(C)** carbohydrate esterases (CEs, n = 25), and **(D)** glycoside hydrolases (GHs n = 343). *S.* sp*raguei* had the highest number of chitin active genes (n = 93), followed by S. *ampiporus* (n = 82), *S. clintonianus* (n = 81), *S. americanus* (n = 77), *S. luteus* (n = 74) and *S. weaverae* (n = 67). The most abundant chitin-active CAZyme families were GH16 (n = 125) and GH18 (n = 110), followed by GH3 (n = 59), and the non-catalytic CBMs CBM5 and CBM50 (LysM) (each with 46).

We identified 68 orthogroups with significant matches to the MEROPS database, totaling 671 individual genes. These included 363 genes in *S.* sp*raguei*, 81 in *S. ampliporus*, 66 in *S. clintonianus*, 64 in *S. americanus*, 51 in *S. luteus*, and 46 in *S. weaverae*, (**Figure 3A**). These included proteases with five distinct catalytic mechanisms including those using aspartic (n = 410), cysteine (n = 12), metallo (n = 82), serine (n = 100), and threonine (n = 67) as key residues. *S.* sp*raguei* had an exceptionally high number of genes encoding aspartic proteases (n = 312, vs. 11–34 in the other species) (**Figure 3A**). These aspartic proteases were dominated by polyporopepsins (**Supplementary Figure S1A**, **Supplementary Table S1**) distributed between a total of 26 orthogroups. Seven of these orthogroups contained a high number of gene copies (up to as 72) and were only present in *S.* sp*raguei* (**Supplementary Figure S1B**).

#### Chitin

In total, we identified 474 genes with putative chitin binding and degradation potential (**Figures 3B-D**) *S.* sp*raguei* had the highest number of chitin active genes (n = 93), followed by S. *ampiporus* (n = 82), *S. clintonianus* (n = 81), *S. americanus* (n = 77), *S. luteus* (n = 74) and *S. weaverae* (n = 67). These genes included the carbohydrate-binding modules (CBMs) CBM5, CBM12, CBM18 and CBM50 (**Figure 3B**), the carbohydrate esterases (CEs) CE4 (Chitin deacetylase and Chitin oligosaccharide deacetylase) and CE9 (N-acetylglucosamine-6-phosphate deacetylase) (**Figure 3C**). We also identified genes encoding chitin active enzymes in the glycoside hydrolase (GH) families GH2 (β-N-acetylhexosaminidase, Exo-β-1,4-glucosaminidase), GH3 (β-N-acetylhexosaminidase, Chitosanase), GH9 (Exo-β-1,4-glucosaminidase), GH16 (Chitin β-1,3/1,6-glucanosyltransferase), GH18 (Chitinase, Chitosanase, Chitin exo-β-1,4-N-acetylglucosaminidase, Chitin exo-β-1,4-N-acetylglucobiosaminidase, Di-N-acetylchitobiase/reducing-end exo-hexosaminidase, [reducing end] exo-chitinase, β-N-acetylhexosaminidase), GH20 (β-1,6-N-acetylglucosaminidase, β-N-acetylhexosaminidase), GH23 (Chitinase), and GH35 (Exo-β-1,4-glucosaminidase) (**Figure 3D**). Auxiliary activity (AA) CAZymes encoding LPMOs active on chitin (families AA10, AA11, and AA15) were not found.

CBMs are non-catalytic proteins that typically co-occur with catalytic enzymes, such as GHs, enhancing their binding affinity and catalytic efficiency (Hartl et al., 2012; Mekasha et al., 2020). CBM5 is typically associated with GH18, increasing the efficiency of chitin degradation (Nagy et al., 2023). CBM5 copy number ranged between 6 in *S. weaverae* and 9 in both *S.* sp*raguei* and *S. ampliporus*. Notably, the species with the lowest number of CMB5 CAZymes were also the lowest in GH18 (**Figures 3B, D**). CBM12 is distantly related to CBM5, and is similarly associated with GH18 (Itoh and Kimoto, 2019). CBM12 occurred in either one or two copies (for *S. clintonianus* and *S. weaverae*). CBM18 binds chitin in association with GH16 (Viborg et al., 2019), and was single-copy in all species. CBM50 (LysM) domains are found attached to various GH families, including GH18 and GH23 (Gruber et al., 2011; López-Sánchez et al., 2024). LysM domains are involved in cleaving both chitin and peptidoglycan, and potentially play additional roles as fungal effectors (Akcapinar et al., 2015). CMB50 displayed the highest copy number in *S. ampliporus* (n = 11), and the lowest in *S. clintonianus* and *S. weaverae* (both with n = 6 copies).

CEs are catalytic enzymes that hydrolyze ester bonds in a variety of substrates. CE4 encodes chitin deacetylase, which is involved in chitin degradation via the chitosan pathway, where chitin is first deacetylated to chitosan before hydrolysis by chitosanases (Veneault-Fourrey et al., 2014). All six *Suillus* species contained 3 copies of CE4, except for *S.* sp*raguei*, which contained 4 copies. GH75 chitosanases are canonically associated with hydrolysis in the chitosan pathway (Cheng et al., 2006), but no GH75 CAZymes were identified. However, other CAZyme families known to encode chitosanases were present, including GH3 and GH18, potentially representing alternative sources of hydrolysis. CE9 encodes N-acetylglucosamine-6-phosphate deacetylase, active on phosphorylated N-acetylglucosamine units (GlcNAc-6-P), which can be derived from chitin degradation products after phosphorylation (Vincent et al., 2004). CE9 displayed no copy number variation, with a single gene present in each species.

GHs are the most abundant and diverse class of CAZymes, with multiple families playing roles in chitin degradation. GH16 CAZYmes bind to a variety of substrates, including fungal β-glucans, xyloglucans, galactans, and chitin, playing diverse roles in both degradation and remodeling (Patel and Free, 2019; Peng et al., 2022). This suite of enzymes incudes chitin β-1,3/1,6-glucanosyltransferase, with roles in chitin modification (Veneault-Fourrey et al., 2014). In this study, GH16 was the most abundant chitin-relevant CAZyme family (n = 125 in total) with copy number variation between 24 (in *S.* sp*raguei*) and 19 (in both *S. ampliporus* and *S. weaverae*). GH18 CAZymes are a widely distributed family of chitinases that occur in most organisms, taking on diverse roles in degradation, remodeling, pathogen defense, and host invasion (Chen et al., 2020). GH18 copy number varies greatly among fungal species, from 1 in *Schizosaccharomyces pombe* to 41 in *Mycogone perniciosa* and is considered to be one of the best predictors of chitin degradation in fungi (Gruber and Seidl-Seiboth, 2012; Yang et al., 2020; Maillard et al., 2023). In this study, GH18 was the second most abundant chitin-relevant CAZyme family (n = 110 in total). GH18 displayed particularly high copy number in *S.* sp*raguei* (n = 28) and *S. ampliporus* (n = 23). Conversely, *S. weaverae* and *S. luteus* contained only 13 copies of GH18. GH3s are a large family of enzymes binding diverse β-glycans as well as chitin. GH3 was the third most abundant chitin-relevant CAZyme family (n = 59 in total), and varied between 8 in *S.* sp*raguei* and 12 in *S. clintonianus*.

### *In vitro* growth assays

N source was the most significant driver of *Suillus* biomass production (p < 2e-16, F = 139.135, accounting for approximately 64% of the total variation), followed by species (p < 0.001, F = 6.949, ~4% of variation) and the interaction between N source and species (p < 0.01, F = 2.398, ~5% of variation). Overall, *S.* sp*raguei* had the highest biomass production producing significantly more biomass than all other species (p < 0.05), followed by *S. luteus* and *S. clintonianus*. *Suillus* grown on ammonium produced significantly more biomass than other treatments (p < 0.0001). Among organic N treatments, protein alone supported significantly higher biomass than the other substrates (p < 0.0001), and protein-mineral complexes produced significantly more biomass than protein-tannin complexes (p < 0.01). All species showed a similar hierarchical response to ammonium treatment with more variable hierarchies among organic N substrates. While BSA generally outperformed the protein-mineral complexes and chitin treatments, the relative performance on protein-mineral, protein-tannin, and chitin varied by species. *S. weaverae* demonstrated the strongest overall response to N source, while *S. americanus* showed the least differentiation among treatments.

**Figure 4:**
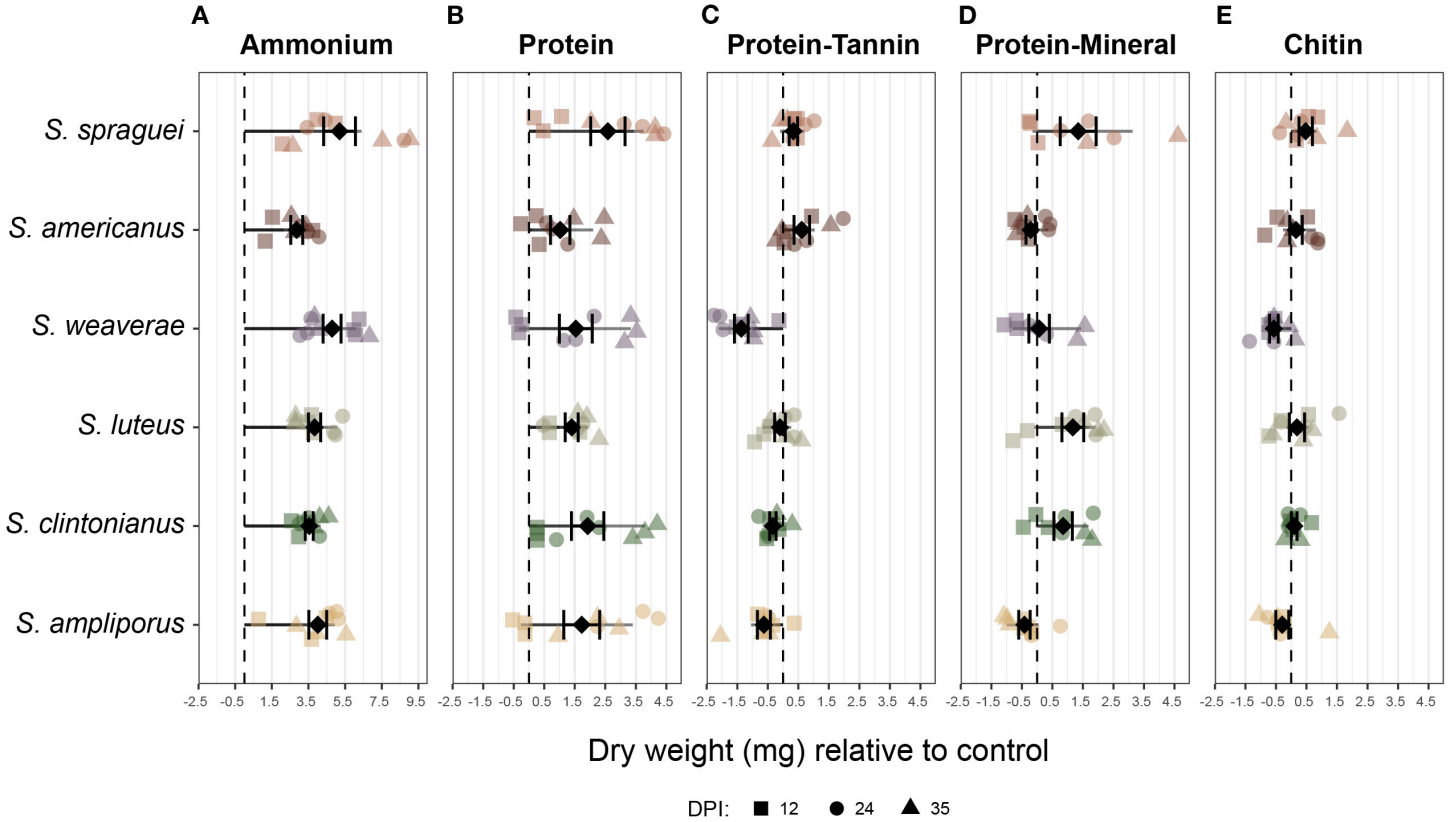
*Biomass production of* Suillus *grown on different nitrogen sources.* Fungal biomass production (dry weight in mg) relative to growth on low ammonium control media for six species of *Suillus* grown on five different N sources: **(A)** Ammonium, **(B)** protein (bovine serum albumin), **(C)** protein-tannin complexes, **(D)** protein-mineral complexes, and **(E)** Chitin. Data points represent individual replicates collected at 12 (square), 24 (circle), and 35 (triangle) days post-inoculation (DPI) at n=3 replicates per time point. Black diamonds with error bars show the mean ± standard error. The dashed vertical line at 0 represents no change in biomass relative to the low ammonium control. N source had the strongest effect on biomass production, with significantly higher growth on ammonium than all other treatments (p < 0.0001). Among organic N sources, protein supported the highest biomass (p < 0.0001), and protein–mineral complexes outperformed protein–tannin complexes (p < 0.01). Across species, *S.* sp*raguei* produced significantly more biomass than all others (p < 0.05). Pairwise comparisons included greater biomass of *S.* sp*raguei* than *S. americanus* on ammonium (p = 0.021) and *S.* sp*raguei* than *S. weaverae* on chitin (p < 0.01).

On ammonium, *S.* sp*raguei* produced significantly more biomass (5.20 mg ± 2.60 SD) than *S. americanus* (2.86 mg ± 0.98) (Tukey’s HSD, p = 0.021), but no other significant differences were detected on ammonium, despite numerical differences in mean biomass production (**Figure 4A**). When grown on protein, *S.* sp*raguei* produced the highest mean biomass (an average of 2.58 mg ± 1.68 SD more than controls), followed by *S. clintonianus* (1.92 mg ± 1.59) and *S. ampliporus* (1.73 mg ± 1.77). However, all six species displayed positive growth on protein, with biomass production not being statistically significantly different among species (**Figure 4B**). On protein complexed to tannin, all species showed poor or negative growth compared to low ammonium controls. *S. americanus* maintained the highest biomass production (0.61 ± 0.76 SD), followed by *S.* sp*raguei* (0.34 ± 0.41 SD), while other species displayed growth inhibition, with *S. weaverae* and *S. ampliporus* exhibiting the strongest negative responses (**Figure 4C**). Species varied considerably in their ability to utilize protein absorbed on mineral surfaces, with *S.* sp*raguei* (1.35 ± 1.66 SD), *S. luteus* (1.17 ± 1.07 SD), and *S. clintonianus* (0.85 ± 0.86 SD) displaying positive growth, *S. weaverae* (0.07 ± 0.95 SD) minimal positive growth and *S. americanus* (-0.21 ± 0.45 SD) and *S. ampliporus* (-0.42 ± 0.56 SD) exhibiting growth inhibition compared to controls (**Figure 4D**). On chitin, *S.* sp*raguei*, *S. luteus*, *S. americanus*, and *S. clintonianus* all displayed slightly positive growth, and *S. ampliporus*, *S. weaverae* displayed slight growth inhibition, with the only statistically significant difference between *S.* sp*raguei* and *S. weaverae* (p < 0.01) (**Figure 4E**).

### *In situ* elemental and isotopic analyses

#### Total N concentration over time

**Figure 5:**
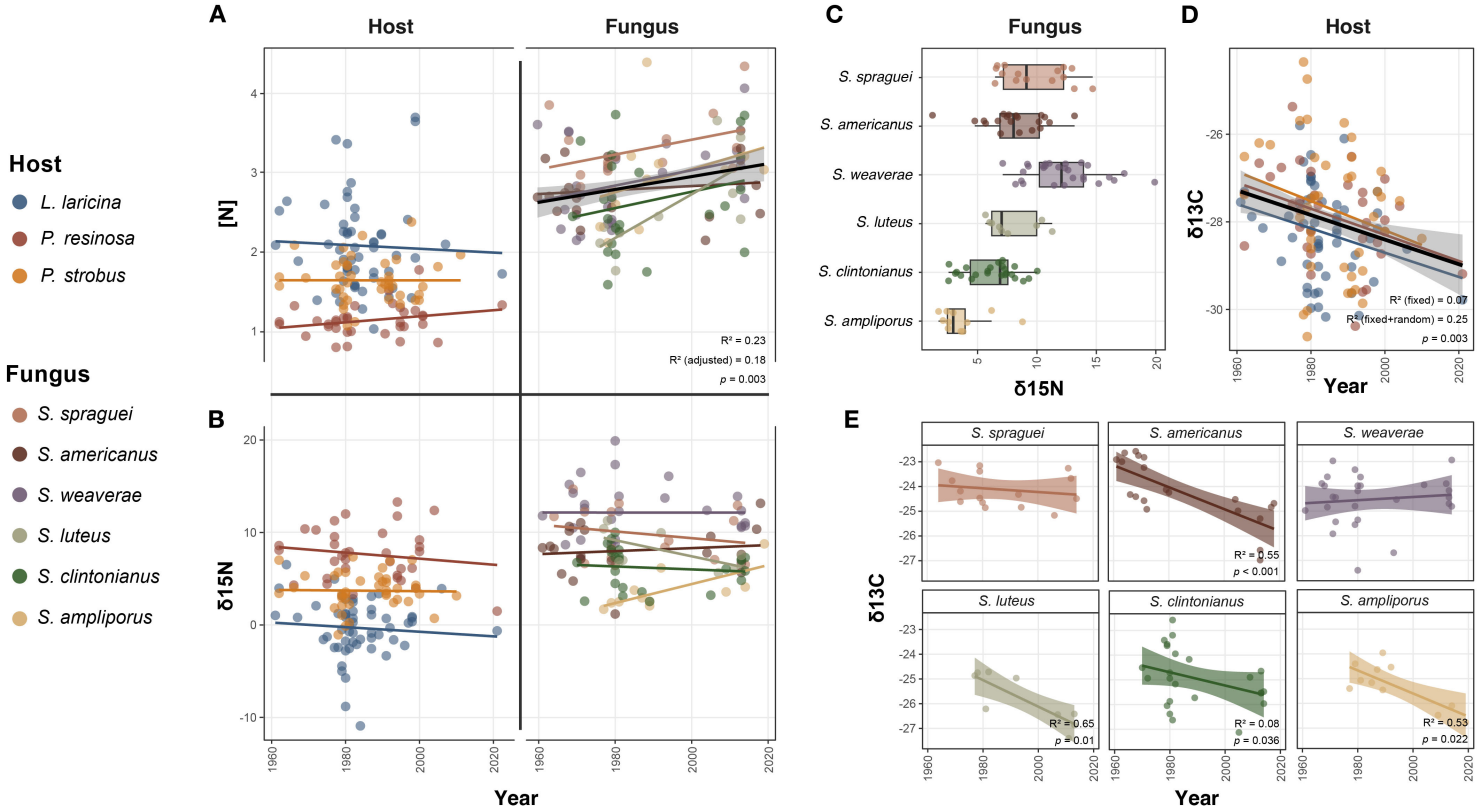
*Isotopic abundance of nitrogen and carbon in historical collections of host trees and* Suillus *fungi.* For all graphs, lines represent the overall linear trend, with shading indicating the 95% confidence interval, and solid black lines representing the overall linear trend. The concentration of total N for **(A)** host and fungus, demonstrated a lack of significant N depletion over time for host trees, and significant increases in N concentration for *Suillus*, with *S.* sp*raguei* demonstrating higher N content than other species. **(B)** Total δ^15^N for host and fungus displayed no significant trends over time, but significant structure by **(C)** fungal species identity. **(D)** Host δ^13^C decreased significantly over time, but was not significantly structured by host species identity. This was in contrast to fungal δ^13^C **(E)**, which decreased significantly for *S. americanus*, *S. ampliporus*, *S. clintonianus*, and *S. luteus*, but did not decrease significantly for *S.* sp*raguei* or *S. weaverae*.

For both host tree needle N concentration and *Suillus* sporocarp N, stepwise selection eliminated the interaction between year and species, and the random effects of county and month, with the model for host N also eliminating time. For host trees, N concentration was best explained by species (**Figure 5A**): *P. resinosa* had the lowest N concentration on average (Coefficient ± SE: 1.16 ± 0.07, t = 16.24, p < 0.0001), followed by *P. strobus* (1.66 ± 0.06, t = 25.90, p < 0.0001), and *L. laricina* (2.09 ± 0.06, t = 37.38, p < 0.0001) (adjusted r² = 0.45). Contrary to expectations, *Suillus* sporocarp N concentrations actually increased over time (0.01 ± 0.003 yr^-1^, t = 3.29, p = 0.0002) with species identity accounting for differences in baseline N concentration (adjusted r² = 0.18). Cumulatively, average sporocarp N concentration increased by approximately 21%, from 2.60% in 1961 (95% CI: 2.30, 2.89) to 3.14% in 2019 (2.84, 3.44).

#### δ^15^N

After stepwise selection, county was preserved as a random effect in the models describing tree needle and sporocarp δ^15^N. In host trees, needle δ15N did not change over time; instead, it was best described by species identity (**Figure 5B**): *P. resinosa* had the highest average δ^15^N (7.57 ± 0.49‰, t = 15.47, p < 0.0001), followed by *P. strobus* (3.77 ± 0.45‰, t = 8.40, p < 0.0001) and *L. laricina* (-0.24 ± 0.41‰, t = -0.59, p = 0.56) (marginal r² = 0.58, conditional r² = 0.64). Sporocarp δ^15^N also did not relate to time; instead, δ^15^N was best explained by species’ identity (marginal r² = 0.50, conditional r² = 0.60) (**Figure 5C**). *S. weaverae* had the highest δ^15^N (12.26 ± 0.56‰, t = 21.77, p < 0.0001), followed by *S.* sp*raguei* (10.12 ± 0.72%, t = 14.04, p < 0.0001), *S. luteus* (8.14 ± 0.89‰, t = 9.19, p < 0.0001), *S. americanus* (8.11± 0.66‰, t = 12.35, p < 0.0001), *S. clintonianus* (6.66 ± 0.62‰, t = 10.70, p < 0.0001) and *S. ampliporus* (3.71 ± 0.80‰, t = 4.62, p < 0.0001).

#### δ^13^C

Needle δ^13^C was best described by year, and declined significantly over time (-0.03 ± 0.01‰ yr^-1^, t = -3.06, p = 0.003), but time alone described only a small portion of the overall variation (marginal R² = 0.071) (**Figure 5D**) Including geographic variation (county) as a random effect (variance = 0.27, p = 0.029) improved model performance considerably (conditional R² = 0.245). Cumulatively, needle δ^13^C declined by 1.73‰ on average, from -27.24‰ in 1961 to -28.97‰ in 2019 (absolute change: -1.73‰ yr^-1^, 95% CI: -2.85‰, -0.61‰), representing a 6.3% decrease (95% CI: -10.4%, -2.2%). Sporocarp δ^13^C was best described by year, species identity, and an interaction between species and year. The effect of time on δ^13^C varied notably between species (**Figure 5E**). While δ^13^C declined significantly in *S. luteus* (-0.054 ± 0.02‰ yr^-1^, t = -2.63, p = 0.010), followed by *S. ampliporus* (-0.047 ± 0.020‰ yr^-1^, t = -2.33, p = 0.022), *S. americanus* (-0.044 ± 0.010‰ yr^-1^, t = -4.51, p < 0.001), and *S. clintonianus* (-0.027 ± 0.013‰ yr^-1^, t = -2.13, p = 0.036), δ^13^C did not change significantly over time in *S.* sp*raguei* (-0.008 ± 0.015‰ yr^-1^, t = -0.52, p = 0.605) or *S. weaverae* (0.006 ± 0.010‰ yr^-1^, t = 0.63, p = 0.527).

#### Difference between cap and stipe δ^15^N

After stepwise selection, the difference between cap and stipe δ^15^N was best explained by year, species identity, their interaction, average sporocarp N, and the difference between cap and stipe N concentrations (adjusted R² = 0.213). While both N concentration and *δ^15^N* concentration were higher in cap tissue than stipe tissue as expected (**Supplementary Figures S2A, B**), δ^15^N difference was negatively related to average sporocarp N concentrations (-0.90 ± 0.33‰, t = -2.69, p = 0.008) (**Supplementary Figure S2C**), and marginally positively related to the difference between cap and stipe N (0.59 ± 0.31‰, t = 1.93, p = 0.056) (**Supplementary Figure S2D**), Temporal trends in δ^15^N difference varied by species: *S. americanus*, *S. ampliporus*, *S. clintonianus*, and *S. weaverae* exhibited no significant temporal trend (p = 0.522, p = 0.188, p = 0.532, p = 0.782, respectively), while *S. luteus* and *S.* sp*raguei* exhibited significant declines in cap-stipe δ^15^N difference (-0.08 ± 0.04‰ yr^-1^, t = -2.16, p = 0.034; -0.09 ± 0.03‰ yr^-1^, t = -3.40, p < 0.001, respectively).

## Discussion

Using genomics, growth assays, and isotopic analyses of historical specimens, we examined N assimilation across six confer-associated *Suillus* species. Our results revealed significant interspecific variation in the genomic capacity for organic N metabolism. Differences in fungal biomass production when grown on different N sources largely corresponded with the abundance of genes involved in accessing these resources, but did not fully account for observed differences in N source utilization. Isotopic patterns further support these findings and indicate that coniferous forests and their fungal symbionts exhibit distinct responses to shifts in N availability compared to broadleaf systems (Michaud et al., 2024).

### Phylogenetic patterns and genomic toolkits for nitrogen acquisition

Phylogenetic placement of the six *Suillus* species agreed with previous analyses (Nguyen et al., 2016; Lofgren et al., 2021) showing clear patterns of host specialization (**Figure 1**). However, phylogenetic relationships did not consistently align with genomic repertoire or N acquisition strategies. For example, while the three *P. strobus* associates (*S.* sp*raguei*, *S. americanus*, and *S. weaverae*) formed a well-supported clade, they exhibited markedly different capacities for organic N assimilation (**Figure 3**). These results were echoed in the genomic analyses of all six species, which revealed notable interspecific variation in the genetic machinery for N acquisition across *Suillus* that did not consistently follow phylogenetic relationships. In contrast, the genes for inorganic N (ammonium and nitrate) were highly conserved and predominantly single-copy (**Figures 2A-C**). In ECM fungi, ammonium uptake occurs via the ammonium importers AMT1, AMT2, and AMT3, characterized in the ECM species *Hebeloma cylindrosporum* (Javelle et al., 2001, 2003), *Amanita muscaria* (Willmann et al., 2007), and *Tuber borchii* (Montanini et al., 2002). While AMT3 is thought to be a low-affinity ammonium transporter, AMT1 and AMT2 function as high affinity transporters, and are subject to N repression (Javelle et al., 2003; Willmann et al., 2007). Here, we were unable to confidently assign the orthogroups to their respective AMT genes, where both orthogroup’s OG0009316 and OG0006824 appeared to be more closely related to the AMT3 reference ortholog HcAmt3, and OG0006295 was more closely related to HcAmt2 (**Figure 2B**). The inability to confidently map the three gene clusters to their corresponding reference sequences highlights both the high sequence similarity between genes in the AMT superfamily, and the need for functional confirmation to distinguish the identity and impact of high- and low-affinity ammonium transporters. Nitrate uptake into fungal tissues is an active process carried out by N transporters, prior to being reduced to nitrite by nitrate reductases and finally to ammonium by nitrite reductase (Takaya, 2002). Compared to other ECM fungal species *Suillus* generally grows well on nitrate, producing both significantly more biomass and NO^-2^ (indicating reductase activity) than many other ECM genera (Nygren et al., 2008). Despite the conserved nature of inorganic N genes across all species, we observed notable differences in ammonium utilization efficiency (**Figure 4A**). These functional differences suggest that variation in gene expression, post-translational modifications, or transporter kinetics may influence N acquisition and subsequent transfer to host trees, even when core genetic machinery appears conserved.

Unlike the highly conserved genes for inorganic N access, we identified substantial differences in the abundance of genes encoding both protein and chitin-degrading enzymes (**Figure 3**). *S.* sp*raguei* had the highest number of GH18 chitinases (28) compared to only 13 in *S. weaverae* and *S. luteus*. This variation is comparable to that observed in previous investigations of intergeneric variation in GH18 abundance across 12 species of ECM fungi which identified 6–22 genes encoding GH18 CAZYmes (Maillard et al., 2023). *S.* sp*raguei* also possessed an exceptional expansion of aspartic proteases (312 in *S.* sp*raguei* compared to 34 in *S. ampliporus*, 22 in *S. clintonianus*, and as low as 11 in *S. weaverae* (**Figure 3**). These aspartic proteases, also known as acid proteases because they are most active under acidic conditions, play diverse roles in both host-interactions and the liberation of organic N, and have previously been shown to be the dominant proteolytic enzyme class enzyme produced by *Suillus* (Theron and Divol, 2014; Rineau et al., 2016). Additional analysis identified these aspartic proteases as polyporopepsins (**Supplementary Figure S1**, **Supplementary Table S2**), secreted enzymes implicated in the liberation of nutrients from complex organic substrates. In *Paxillus involutus* (also a member of the order Boletales), polyporopepsins are the most highly upregulated enzymes during organic N assimilation (Shah et al., 2013), and among the most highly upregulated enzymes during late-stage carbon starvation (Ellström et al., 2015).

### Functional performance and genomic correspondence

The interspecific differences in N metabolism identified using genomics corresponded with functional performance in the laboratory bioassays, where all species grew well on ammonium but showed distinct patterns of biomass production on organic N sources that generally aligned with their genomic potential. On protein, *S.* sp*raguei* produced the greatest amount of biomass, followed by *S. clintonianus* and *S. ampliporus* (**Figure 4B**). Most species showed poor or negative growth relative to controls on protein-tannin complexes (**Figure 4C**), suggesting limitations in their ability to access protein bound to phenolic compounds. These results are in agreement with previous work suggesting that phenolics inhibit the mobilization of N for many ECM fungi (Bending and Read, 1996). In contrast, most species maintained positive growth on protein absorbed on to minerals, though individual responses were highly species-specific (**Figure 4D**). These results both confirm the ability of certain ECM fungi to acquire organic N from mineral-associated proteins (Wang et al., 2021), and indicates that while protein bound to minerals may be more accessible than protein bound to phenolic compounds, the ability to access mineral-bound N varies considerably among species. On chitin *S.* sp*raguei* again demonstrated the greatest biomass production with *S. luteus*, *S. americanus*, and *S. clintonianus* also displaying positive growth relative to controls, and *S. ampliporus* and *S. weaverae* displaying growth inhibition (**Figure 4E**). Notably, these trends were largely reflected in the abundance of chitin-active genes, where *S. weaverae* displayed the lowest number of chitin-active genes overall, and the worst overall growth when grown on chitin- a trend that was reversed for *S.* sp*raguei.* These results may have been driven by the GH16 family, where *S. ampliporus* and *S. weaverae* displayed the lowest number of GH16 chitinases (19 each) in the genomic analyses, and *S.* sp*raguei* the highest (with 24).

### Historical evidence for divergent nitrogen acquisition strategies

ECM fungi typically exhibit higher δ^15^N and lower δ^13^C than saprotrophic species, reflecting their use of ^13^C-depleted host photosynthate and ^15^N-enriched N sources. The ^15^N enrichment in ECM fungi may result from several processes: preferential transfer of ^14^N to host trees, acquisition of ^15^N-enriched N from deeper soil profiles, or sequestration of ^14^N in belowground mycelia that concentrates ^15^N in sporocarp proteins (Hobbie and Ouimette, 2009; Hobbie and Agerer, 2010; Hobbie et al., 2012). In this study, we found *Suillus* exhibited a range of δ^15^N values that fell within those previously reported for ECM fungi (Mayor et al., 2009). The higher concentrations of both total N and δ^15^N in cap tissue than in stipe tissue (**Supplementary Figures S2A, B**), matched expectations of tissue-specific protein concentration and isotopic fractionation (Hobbie et al., 2012). This trend was also reflected in the marginally positive relationship between differences in cap-stipe total N concentration and differences in cap-stipe δ^15^N (**Supplementary Figure S2C**), indicating that ^15^N enrichment corresponds with greater protein allocation to caps. However, the significantly negative relationship between overall sporocarp N status and differences in cap-stipe δ^15^N suggests that N-replete fungi exhibit reduced isotopic fractionation (**Supplementary Figure S2D**), possibly reflecting more efficient transport or reduced discrimination against δ^15^N when N availability is high.

Our isotopic analyses of historical specimens revealed complex N dynamics that challenge general predictions about terrestrial oligotrophication in coniferous forests. Contrary to expectations, total fungal N concentration increased significantly over time, with sporocarp N rising by approximately 21% from 1961 to 2019, while host plant N concentrations remained relatively stable and species-specific (**Figure 5A**). Strikingly, this finding contrasts sharply with a recent study from the same region that documented declining foliar N in broadleaf trees along with declining ECM sporocarp N over time (Michaud et al., 2024). Such contrasting N trajectories between broadleaf and conifer species have been documented elsewhere, including in Europe (Penuelas et al., 2020), suggesting fundamental differences in how these forest types respond to changing N availability. In particular, conifers may be more responsive to N deposition than broadleaf trees, potentially explaining this difference. Additionally, *Suillus* species exhibit ecological traits that distinguish them from the genera sampled in Michaud et al. (2024), including greater sensitivity to N deposition, high host-specificity, and unique mycelial morphology thought to be related to organic N acquisition (Lilleskov et al., 2011). The observed increase in *Suillus* sporocarp N despite proposed oligotrophication therefore suggests that these fungi and their coniferous hosts are responding differently to changing N regimes than broadleaf forest systems, potentially reflecting greater efficiency in organic N acquisition or both responses to and influences on the availability of different N pools across forest types.

### Species-specific strategies and resource partitioning

Beyond these temporal trends in total N content, our isotopic analyses also provided detailed evidence of species-specific N acquisition strategies, with both host trees and fungi showing stable fractionation patterns over time but distinct species-level differences (**Figures 5B, C**). The carbon isotope data revealed divergent responses to changing atmospheric conditions, with host needles showing a steady decline in δ^13^C over time consistent with the Suess effect (**Figure 5D**), while fungal responses varied markedly by species. In line with the δ^15^N results, four species of *Suillus* (*S. luteus*, *S. ampliporus*, *S. americanus*, and *S. clintonianus*) exhibited significant declines in δ^13^C over time, mirroring the Suess effect and suggesting reliance on recently fixed carbon sources (**Figure 5E**). In contrast, *S.* sp*raguei* and *S. weaverae* maintained stable δ^13^C values despite ongoing atmospheric δ^13^C decline, indicating greater utilization of older soil organic matter pools for both C and N acquisition. While the increased incorporation of δ^13^C likely reflects the use of organic N sources (which contain C-N bonds) as shown in *Paxillus involutus* (Akroume et al., 2019), it is possible that these patterns are influenced by the direct uptake of C, where stable δ^13^C in *S.* sp*raguei* and *S. weaverae* could indicate the some degree of autonomous exploitation of carbon resources in soil organic matter reserves.

While *S. weaverae* and *S.* sp*raguei* exhibited high δ^15^N values and stable δ^13^C values over time consistent with preferential access to older organic N pools, *S. americanus* displayed intermediate δ^15^N and a decrease in δ^13^C over time. Despite sharing the same host, these species appear to have evolved diverse N acquisition strategies indicating that differences in N use may have evolved in response to local ecological factors independent of host association. Soil N in ECM forests becomes progressively ^15^N-enriched with depth, and this functional diversity may reflect resource partitioning strategies that reduce interspecific competition, potentially enabling ECM fungi to specialize on different soil profiles and associated N pools, as demonstrated for *Rhizopogon* (Mujic et al., 2015). Such specialization on different N sources may have numerous downstream effects on metabolic function, as evidenced by *S. americanus* being more competitive than *S.* sp*raguei* (Kennedy et al., 2020), potentially reflecting more efficient resource incorporation or tighter host associations. These results also highlight complex relationships between host and fungal N status that may interact synergistically. For example, while *L. laricina* maintained higher foliar N concentrations over time compared to *P. strobus* and *P. resinosa*, this pattern was reversed for δ^15^N (**Figures 5A-C**). These results may reflect fundamental differences in growth environment such as access to recently fixed or mineralized N sources in wetlands, or differences in the physiology, or fractionation patterns of host species. The low δ^15^N in the two *L. laricina* associates, *S. ampliporus* and *S. clintonianus*, may not only influence the low δ^15^N of their host (*L. laricina* needle δ^15^N was approximately 7.8‰ and 4.0‰ lower in δ^15^N than *P. resinosa* and *P. strobus*, respectively), but may also be influenced by the host, via the breakdown and assimilation of host litter which contains low δ^15^N. The mechanisms underlying these diverse N acquisition strategies and host-fungal interactions likely depend on soil chemical conditions such as pH, which may influence both organic and inorganic N accessibility.

For organic N, pH mediates the activity of acid-dependent enzymes like polyporopepsins, identified in high copy number in *S.* sp*raguei*. Simultaneously, pH affects inorganic N access, as demonstrated by *P. involutus* where optimal ammonium uptake occurs between pH 4 and 5.5 (Javelle et al., 1999). Mechanistically, fungal ammonium assimilation via AMT transporters such as those identified in this study, rely on proton motive force through secondary active transport (Williamson et al., 2024). Although the relative contributions of proton gradient versus membrane potential remain unclear, acidic environments likely influence the efficiency of ammonium transport, particularly if proton motive force is primarily driven by proton gradient. Taken together, these patterns indicate that pH has the potential to shape both organic and inorganic N assimilation in ECM fungi (Fernandez and See, 2025), but the mechanisms and ecological consequences of these effects require further investigation.

## Future directions and conclusions

Our integration of genomics, bioassays, and isotopic analyses provides unprecedented insight into N metabolism in an important model genus for ECM ecology (Lofgren et al., 2024), allowing us to compare genetic potential with both functional performance and observed ecological patterns across temporal scales. While our genomic analyses identified patterns of gene presence/absence and copy number variation, future transcriptomic studies under varying N conditions are needed to validate gene activity and provide mechanistic links between genotype and phenotype. Further, while our bioassays included protein-tannin and protein-mineral complexes in an attempt to better represent the complexity of soil organic matter, these had a limited and inconsistent effects on organic N assimilation. Additional experiments with a broader spectrum of naturally-derived organic N sources, inorganic:organic N ratios, and environmental parameters would strengthen connections between laboratory-based results and realized N use under field conditions.

The divergent N acquisition strategies demonstrated by different *Suillus* species represent a dimension of ECM functional diversity with significant ecological implications. Differences across closely related species facilitate access to distinct pools of soil N, potentially reducing competition between co-occurring species while maintaining host access to diverse N sources. This functional diversity may prove critical for forest resilience as global change and anthropogenic activities continue to alter terrestrial N dynamics. The loss of specific ECM species could impair specific N acquisition pathways, potentially limiting nutrient access for host trees under changing conditions. Moreover, the divergent responses to N availability we observed over time indicate that forest ecosystems may respond to climate change and N deposition in complex, species-specific ways not captured by current biogeochemical models. As atmospheric CO_2_ continues to rise and N deposition patterns shift, the specialized metabolic capacities of different ECM fungi may become increasingly important determinants of forest productivity, carbon sequestration, and overall ecosystem function, highlighting the need to incorporate fungal functional diversity into predictions of forest responses to global change.

Collectively, this interspecific investigation reveals the extent of ECM functional diversity, demonstrating that even within a single genus, fungi employ fundamentally different N acquisition strategies with implications for forest ecosystem resilience. However, because our phylogenetically constrained dataset represents only a small fraction of ECM diversity, expanding this framework to encompass a greater taxonomic diversity (on both the plant and fungal side of symbiosis) will enable quantitative modeling that mechanistically links genomic potential, functional performance, and ecosystem-level N cycling.

## Data Availability

The datasets presented in this study can be found in online repositories. The names of the repository/repositories and accession numbers can be found below: https://doi.org/10.5281/zenodo.15865691, 10.5281/zenodo.15865691.

## Supplementary material

Supplementary Figure 1
*Distribution and conservation of polyporopepsins.* **(A)** Presence/absence conservation of orthogroups encoding polyporopepsins across *Suillus* species. **(B)** Copy number variation within individual polyporopepsin orthogroups, highlighting multiple gene amplifications in *S.* sp*raguei*.

Supplementary Figure 2
*Difference between cap and stipe δ^15^N.* (N) concentration A) and δ^15^N concentration **(B)** were higher in cap tissue than stipe tissue. The difference between cap and stipe δ^15^N **(C)**, was negatively related to average sporocarp N concentration and marginally positively related to the difference between cap and stipe N concentration **(D)**. All graphs are color coded by species. Lines in **(A, B)** connecting stipe-cap datapoints represent samples taken from the same fruitbody. In **(C, D)** shading indicates the 95% confidence interval, and solid black lines representing the overall linear trend.

Supplementary Table 1 Metadata for plant and fungal herbaria samples used for isotopic analysis.

Supplementary Table 2 Distribution and annotation of aspartic proteases across six species of *Suillus*.

## References

[B1] Adamczyk B. Simon J. Kitunen V. Adamczyk S. Smolander A. (2017). Tannins and their complex interaction with different organic nitrogen compounds and enzymes: old paradigms versus recent advances. ChemistryOpen 6, 610–614., PMID: 29046854 10.1002/open.201700113 PMC5641916

[B2] Akcapinar G. B. Kappel L. Sezerman O. U. Seidl-Seiboth V. (2015). Molecular diversity of LysM carbohydrate-binding motifs in fungi. Curr. Genet. 61, 103–113. doi: 10.1007/s00294-014-0471-9, PMID: 25589417 PMC4392113

[B3] Akroume E. Maillard F. Bach C. Hossann C. Brechet C. Angeli N.. (2019). First evidences that the ectomycorrhizal fungus *Paxillus involutus* mobilizes nitrogen and carbon from saprotrophic fungus necromass. Environ. Microbiol. 21, 197–208. doi: 10.1111/1462-2920.14440, PMID: 30307107

[B4] Arnolds E. (1991). Decline of ectomycorrhizal fungi in Europe. Agriculture Ecosyst. Environ. 35, 209–244. doi: 10.1016/0167-8809(91)90052-Y

[B5] Bassett K. R. Östlund L. Gundale M. J. Fridman J. Jämtgård S. (2023). Forest inventory tree core archive reveals changes in boreal wood traits over seven decades. Sci. total Environ. 900, 165795. doi: 10.1016/j.scitotenv.2023.165795, PMID: 37499833

[B6] Belmecheri S. Lavergne A. (2020). Compiled records of atmospheric CO2 concentrations and stable carbon isotopes to reconstruct climate and derive plant ecophysiological indices from tree rings. Dendrochronologia 63, 125748. doi: 10.1016/j.dendro.2020.125748

[B7] Bending G. D. Read D. J. (1996). Nitrogen mobilization from protein-polyphenol complex by ericoid and ectomycorrhizal fungi. Soil Biol. Biochem. 28, 1603–1612. doi: 10.1016/S0038-0717(96)00258-1

[B8] CAZypedia Consortium (2018). Ten years of CAZypedia: a living encyclopedia of carbohydrate-active enzymes. Glycobiology 28, 3–8. doi: 10.1093/glycob/cwx089, PMID: 29040563

[B9] Chalot M. Brun A. (1998). Physiology of organic nitrogen acquisition by ectomycorrhizal fungi and ectomycorrhizas. FEMS Microbiol. Rev. 22, 21–44. doi: 10.1111/j.1574-6976.1998.tb00359.x, PMID: 9640645

[B10] Chen W. Jiang X. Yang Q. (2020). Glycoside hydrolase family 18 chitinases: The known and the unknown. Biotechnol. Adv. 43, 107553. doi: 10.1016/j.biotechadv.2020.107553, PMID: 32439576

[B11] Cheng C.-Y. Chang C.-H. Wu Y.-J. Li Y.-K. (2006). Exploration of glycosyl hydrolase family 75, a chitosanase from *Aspergillus fumigatus* . J. Biol. Chem. 281, 3137–3144. doi: 10.1074/jbc.M512506200, PMID: 16330537

[B12] Craine J. M. Elmore A. J. Wang L. Aranibar J. Bauters M. Boeckx P.. (2018). Isotopic evidence for oligotrophication of terrestrial ecosystems. Nat. Ecol. Evol. 2, 1735–1744. doi: 10.1038/s41559-018-0694-0, PMID: 30349095

[B13] Drula E. Garron M.-L. Dogan S. Lombard V. Henrissat B. Terrapon N. (2022). The carbohydrate-active enzyme database: functions and literature. Nucleic Acids Res. 50, D571–D577. doi: 10.1093/nar/gkab1045, PMID: 34850161 PMC8728194

[B14] Ellström M. Shah F. Johansson T. Ahrén D. Persson P. Tunlid A. (2015). The carbon starvation response of the ectomycorrhizal fungus *Paxillus involutus* . FEMS Microbiol. Ecol. 91, fiv027. doi: 10.1093/femsec/fiv027, PMID: 25778509 PMC4434801

[B15] Emms D. M. Kelly S. (2019). OrthoFinder: phylogenetic orthology inference for comparative genomics. Genome Biol. 20, 238. doi: 10.1186/s13059-019-1832-y, PMID: 31727128 PMC6857279

[B16] Fernandez C. W. See C. R. (2025). The pH influence on ectomycorrhizal nitrogen acquisition and decomposition. New Phytol. 246, 867–875. doi: 10.1111/nph.70021, PMID: 40065484 PMC11982800

[B17] Fries N. Serck-Hanssen K. Dimberg L. H. Theander O. (1987). Abietic acid, and activator of basidiospore germination in ectomycorrhizal species of the genus Suillus (Boletaceae). Exp. mycology 11, 360–363. doi: 10.1016/0147-5975(87)90024-7

[B18] Gao W. Wang Q. Li N. Wang R. Zhang X. Yin H. (2024). Nitrogen addition enhances stable soil carbon accumulation during ectomycorrhizal hyphae decomposition. Plant Soil, 511, 1–14. doi: 10.1007/s11104-024-07004-y

[B19] Grigoriev I. V. Nikitin R. Haridas S. Kuo A. Ohm R. Otillar R.. (2014). MycoCosm portal: gearing up for 1000 fungal genomes. Nucleic Acids Res. 42, D699–D704. doi: 10.1093/nar/gkt1183, PMID: 24297253 PMC3965089

[B20] Groffman P. M. Driscoll C. T. Durán J. Campbell J. L. Christenson L. M. Fahey T. J.. (2018). Nitrogen oligotrophication in northern hardwood forests. Biogeochemistry 141, 523–539. doi: 10.1007/s10533-018-0445-y

[B21] Gruber S. Seidl-Seiboth V. (2012). Self versus non-self: fungal cell wall degradation in *Trichoderma* . Microbiol. (Reading England) 158, 26–34. doi: 10.1099/mic.0.052613-0, PMID: 21873410

[B22] Gruber S. Vaaje-Kolstad G. Matarese F. López-Mondéjar R. Kubicek C. P. Seidl-Seiboth V. (2011). Analysis of subgroup C of fungal chitinases containing chitin-binding and LysM modules in the mycoparasite *Trichoderma atroviride* . Glycobiology 21, 122–133. doi: 10.1093/glycob/cwq142, PMID: 20843785

[B23] Hartl L. Zach S. Seidl-Seiboth V. (2012). Fungal chitinases: diversity, mechanistic properties and biotechnological potential. Appl. Microbiol. Biotechnol. 93, 533–543. doi: 10.1007/s00253-011-3723-3, PMID: 22134638 PMC3257436

[B24] Hasselquist N. J. Högberg P. (2014). Dosage and duration effects of nitrogen additions on ectomycorrhizal sporocarp production and functioning: an example from two N-limited boreal forests. Ecol. Evol. 4, 3015–3026. doi: 10.1002/ece3.1145, PMID: 25247059 PMC4161175

[B25] Hobbie E. A. Agerer R. (2010). Nitrogen isotopes in ectomycorrhizal sporocarps correspond to belowground exploration types. Plant Soil 327, 71–83. doi: 10.1007/s11104-009-0032-z

[B26] Hobbie E. A. Chen J. Hasselquist N. J. (2019). Fertilization alters nitrogen isotopes and concentrations in ectomycorrhizal fungi and soil in pine forests. Fungal Ecol. 39, 267–275. doi: 10.1016/j.funeco.2018.12.013

[B27] Hobbie E. A. Högberg P. (2012). Nitrogen isotopes link mycorrhizal fungi and plants to nitrogen dynamics. New Phytol. 196, 367–382. doi: 10.1111/j.1469-8137.2012.04300.x, PMID: 22963677

[B28] Hobbie E. A. Ouimette A. P. (2009). Controls of nitrogen isotope patterns in soil profiles. Biogeochemistry 95, 355–371. doi: 10.1007/s10533-009-9328-6

[B29] Hobbie E. A. Sánchez F. S. Rygiewicz P. T. (2012). Controls of isotopic patterns in saprotrophic and ectomycorrhizal fungi. Soil Biol. Biochem. 48, 60–68. doi: 10.1016/j.soilbio.2012.01.014

[B30] Itoh T. Kimoto H. (2019). Bacterial chitinase system as a model of chitin biodegradation. Adv. Exp. Med. Biol. 1142, 131–151. doi: 10.1007/978-981-13-7318-3_7, PMID: 31102245

[B31] Javelle A. Chalot M. Söderström B. Botton B. (1999). Ammonium and methylamine transport by the ectomycorrhizal fungus *Paxillus involutus* and ectomycorrhizas. FEMS Microbiol. Ecol. 30, 355–366. doi: 10.1111/j.1574-6941.1999.tb00663.x, PMID: 10568844

[B32] Javelle A. Morel M. Rodríguez-Pastrana B.-R. Botton B. André B. Marini A.-M.. (2003). Molecular characterization, function and regulation of ammonium transporters (Amt) and ammonium-metabolizing enzymes (GS, NADP-GDH) in the ectomycorrhizal fungus Hebeloma cylindrosporum: Ammonium assimilation in *Hebeloma cylindrosporum* . Mol. Microbiol. 47, 411–430. doi: 10.1046/j.1365-2958.2003.03303.x, PMID: 12519192

[B33] Javelle A. Rodríguez-Pastrana B. R. Jacob C. Botton B. Brun A. André B.. (2001). Molecular characterization of two ammonium transporters from the ectomycorrhizal fungus *Hebeloma cylindrosporum* . FEBS Lett. 505, 393–398. doi: 10.1016/S0014-5793(01)02802-2, PMID: 11576535

[B34] Jilling A. Keiluweit M. Contosta A. R. Frey S. Schimel J. Schnecker J. (2018). Minerals in the rhizosphere: overlooked mediators of soil nitrogen availability to plants and microbes. Biogeochemistry 139, 103–122.

[B35] Jörgensen K. Clemmensen K. E. Fransson P. Manzoni S. Wallander H. Lindahl B. D. (2025). A trait spectrum linking nitrogen acquisition and carbon use of ectomycorrhizal fungi. New Phytol. 246, 2425–2434. doi: 10.1111/nph.70129, PMID: 40186423 PMC12095993

[B36] Kalyaanamoorthy S. Minh B. Q. Wong T. K. F. von Haeseler A. Jermiin L. S. (2017). ModelFinder: fast model selection for accurate phylogenetic estimates. Nat. Methods 14, 587–589. doi: 10.1038/nmeth.4285, PMID: 28481363 PMC5453245

[B37] Katoh K. Standley D. M. (2013). MAFFT multiple sequence alignment software version 7: improvements in performance and usability. Mol. Biol. Evol. 30, 772–780. doi: 10.1093/molbev/mst010, PMID: 23329690 PMC3603318

[B38] Kemppainen M. J. Alvarez Crespo M. C. Pardo A. G. (2010). fHANT-AC genes of the ectomycorrhizal fungus *Laccaria bicolor* are not repressed by l-glutamine allowing simultaneous utilization of nitrate and organic nitrogen sources. Environ. Microbiol. Rep. 2, 541–553. doi: 10.1111/j.1758-2229.2009.00111.x, PMID: 23766224

[B39] Kennedy P. G. Gagne J. Perez-Pazos E. Lofgren L. A. Nguyen N. H. (2020). Does fungal competitive ability explain host specificity or rarity in ectomycorrhizal symbioses? PLoS One 15, e0234099. doi: 10.1371/journal.pone.0234099, PMID: 32810132 PMC7433872

[B40] Knicker H. Schmidt M. W. I. Kögel-Knabner I. (1999). The structure of organic nitrogen in particle size fractions determined by 15N CPMAS NMR. In: Effect of Mineral-Organic-Microorganism Interactions on Soil and Freshwater Environments. Boston, MA, Springer US, 143–149.

[B41] Kohler A. Kuo A. Nagy L. G. Morin E. Barry K. W. Buscot F.. (2015). Convergent losses of decay mechanisms and rapid turnover of symbiosis genes in mycorrhizal mutualists. Nat. Genet. 47, 410–415. doi: 10.1038/ng.3223, PMID: 25706625

[B42] Koide R. T. Courty P.-E. Garbaye J. (2007). Research perspectives on functional diversity in ectomycorrhizal fungi. New Phytol. 174, 240–243. doi: 10.1111/j.1469-8137.2007.01987.x, PMID: 17388886

[B43] Kranabetter J. M. Harman-Denhoed R. Hawkins B. J. (2019). Saprotrophic and ectomycorrhizal fungal sporocarp stoichiometry (C: N: P) across temperate rainforests as evidence of shared nutrient constraints among symbionts. New Phytol. 221, 482–492. doi: 10.1111/nph.15380, PMID: 30084239

[B44] Levasseur A. Drula E. Lombard V. Coutinho P. M. Henrissat B. (2013). Expansion of the enzymatic repertoire of the CAZy database to integrate auxiliary redox enzymes. Biotechnol. Biofuels 6, 41. doi: 10.1186/1754-6834-6-41, PMID: 23514094 PMC3620520

[B45] Lilleskov E. A. Hobbie E. A. Horton T. R. (2011). Conservation of ectomycorrhizal fungi: exploring the linkages between functional and taxonomic responses to anthropogenic N deposition. Fungal Ecol. 4, 174–183. doi: 10.1016/j.funeco.2010.09.008

[B46] Lilleskov E. A. Kuyper T. W. Bidartondo M. I. Hobbie E. A. (2019). Atmospheric nitrogen deposition impacts on the structure and function of forest mycorrhizal communities: A review. Environ. pollut. (Barking Essex: 1987) 246, 148–162. doi: 10.1016/j.envpol.2018.11.074, PMID: 30543941

[B47] Lofgren L. Nguyen N. H. Kennedy P. G. Pérez-Pazos E. Fletcher J. Liao H.-L.. (2024). *Suillus*: an emerging model for the study of ectomycorrhizal ecology and evolution. New Phytol. 242, 1448–1475. doi: 10.1111/nph.19700, PMID: 38581203 PMC11045321

[B48] Lofgren L. A. Nguyen N. H. Vilgalys R. Ruytinx J. Liao H.-L. Branco S.. (2021). Comparative genomics reveals dynamic genome evolution in host specialist ectomycorrhizal fungi. New phytologist 230, 774–792. doi: 10.1111/nph.17160, PMID: 33355923 PMC7969408

[B49] López-Sánchez R. Rebollar E. A. Gutiérrez-Ríos R. M. Garciarrubio A. Juarez K. Segovia L. (2024). Metagenomic analysis of carbohydrate-active enzymes and their contribution to marine sediment biodiversity. World J. Microbiol. Biotechnol. 40, 95. doi: 10.1007/s11274-024-03884-5, PMID: 38349445 PMC10864421

[B50] Lucic E. Fourrey C. Kohler A. Martin F. Chalot M. Brun-Jacob A. (2008). A gene repertoire for nitrogen transporters in *Laccaria bicolor* . New Phytol. 180, 343–364. doi: 10.1111/j.1469-8137.2008.02580.x, PMID: 18665901

[B51] Maillard F. Didion M. Fauchery L. Bach C. Buée M. (2018). N-Acetylglucosaminidase activity, a functional trait of chitin degradation, is regulated differentially within two orders of ectomycorrhizal fungi: Boletales and Agaricales. Mycorrhiza 28, 391–397. doi: 10.1007/s00572-018-0833-0, PMID: 29654366

[B52] Maillard F. Kohler A. Morin E. Hossann C. Miyauchi S. Ziegler-Devin I.. (2023). Functional genomics gives new insights into the ectomycorrhizal degradation of chitin. New Phytol. 238, 845–858. doi: 10.1111/nph.18773, PMID: 36702619

[B53] Mason R. E. Craine J. M. Lany N. K. Jonard M. Ollinger S. V. Groffman P. M.. (2022). Evidence, causes, and consequences of declining nitrogen availability in terrestrial ecosystems. Sci. (New York N.Y.) 376, eabh3767. doi: 10.1126/science.abh3767, PMID: 35420945

[B54] Mayor J. R. Schuur E. A. G. Henkel T. W. (2009). Elucidating the nutritional dynamics of fungi using stable isotopes. Ecol. Lett. 12, 171–183. doi: 10.1111/j.1461-0248.2008.01265.x, PMID: 19049511

[B55] McLauchlan K. K. Ferguson C. J. Wilson I. E. Ocheltree T. W. Craine J. M. (2010). Thirteen decades of foliar isotopes indicate declining nitrogen availability in central North American grasslands. New Phytol. 187, 1135–1145. doi: 10.1111/j.1469-8137.2010.03322.x, PMID: 20553396

[B56] McLauchlan K. K. Gerhart L. M. Battles J. J. Craine J. M. Elmore A. J. Higuera P. E.. (2017). Centennial-scale reductions in nitrogen availability in temperate forests of the United States. Sci. Rep. 7, 7856. doi: 10.1038/s41598-017-08170-z, PMID: 28798386 PMC5552780

[B57] Mekasha S. Tuveng T. R. Askarian F. Choudhary S. Schmidt-Dannert C. Niebisch A.. (2020). A trimodular bacterial enzyme combining hydrolytic activity with oxidative glycosidic bond cleavage efficiently degrades chitin. J. Biol. Chem. 295, 9134–9146. doi: 10.1074/jbc.RA120.013040, PMID: 32398257 PMC7335802

[B58] Michaud T. J. Cline L. C. Hobbie E. A. Gutknecht J. L. M. Kennedy P. G. (2024). Herbarium specimens reveal that mycorrhizal type does not mediate declining temperate tree nitrogen status over a century of environmental change. New Phytol. 242, 1717–1724. doi: 10.1111/nph.19452, PMID: 38073143

[B59] Montanini B. Moretto N. Soragni E. Percudani R. Ottonello S. (2002). A high-affinity ammonium transporter from the mycorrhizal ascomycete *Tuber borchii* . Fungal Genet. biology: FG B 36, 22–34. doi: 10.1016/S1087-1845(02)00001-4, PMID: 12051892

[B60] Mujic A. B. Durall D. M. Spatafora J. W. Kennedy P. G. (2015). Competitive avoidance not edaphic specialization drives vertical niche partitioning among sister species of ectomycorrhizal fungi. New Phytol. 209, 1174–1183. doi: 10.1111/nph.13677, PMID: 26391726

[B61] Nagy L. G. Vonk P. J. Künzler M. Földi C. Virágh M. Ohm R. A.. (2023). Lessons on fruiting body morphogenesis from genomes and transcriptomes of *Agaricomycetes* . Stud. mycology 104, 1–85. doi: 10.3114/sim.2022.104.01, PMID: 37351542 PMC10282164

[B62] Nguyen N. H. Vellinga E. C. Bruns T. D. Kennedy P. G. (2016). Phylogenetic assessment of global Suillus ITS sequences supports morphologically defined species and reveals synonymous and undescribed taxa. Accepted to Mycologia. 108, 1216–28. doi: 10.3852/16-106, PMID: 27760855

[B63] Nicolás C. Martin-Bertelsen T. Floudas D. Bentzer J. Smits M. Johansson T.. (2019). The soil organic matter decomposition mechanisms in ectomycorrhizal fungi are tuned for liberating soil organic nitrogen. ISME J. 13, 977–988. doi: 10.1038/s41396-018-0331-6, PMID: 30538275 PMC6461840

[B64] Nygren C. M. R. Eberhardt U. Karlsson M. Parrent J. L. Lindahl B. D. Taylor A. F. S. (2008). Growth on nitrate and occurrence of nitrate reductase-encoding genes in a phylogenetically diverse range of ectomycorrhizal fungi. New Phytol. 180, 875–889. doi: 10.1111/j.1469-8137.2008.02618.x, PMID: 18783355

[B65] Op De Beeck M. Troein C. Peterson C. Persson P. Tunlid A. 04 (2018). Fenton reaction facilitates organic nitrogen acquisition by an ectomycorrhizal fungus. New Phytol. 218, 335–343. doi: 10.1111/nph.14971, PMID: 29297591 PMC5873446

[B66] Patel P. K. Free S. J. (2019). The genetics and biochemistry of cell wall structure and synthesis in *Neurospora crassa*, a model filamentous fungus. Front. Microbiol. 10, 2294. doi: 10.3389/fmicb.2019.02294, PMID: 31649638 PMC6796803

[B67] Pena R. Tibbett M. (2024). Mycorrhizal symbiosis and the nitrogen nutrition of forest trees. Appl. Microbiol. Biotechnol. 108, 461. doi: 10.1007/s00253-024-13298-w, PMID: 39249589 PMC11384646

[B68] Peng L. Zhang Y. Druzhinina I. S. Kubicek C. P. Wang Y. Zhu Z.. (2022). A facultative ectomycorrhizal association is triggered by organic nitrogen. Curr. biology: CB 32, 5235–5249.e7. doi: 10.1016/j.cub.2022.10.054, PMID: 36402137

[B69] Penuelas J. Fernández-Martínez M. Vallicrosa H. Maspons J. Zuccarini P. Carnicer J.. (2020). Increasing atmospheric CO2 concentrations correlate with declining nutritional status of European forests. Commun. Biol. 3, 125. doi: 10.1038/s42003-020-0839-y, PMID: 32170162 PMC7070084

[B70] Rawlings N. D. Barrett A. J. Thomas P. D. Huang X. Bateman A. Finn R. D. (2018). The MEROPS database of proteolytic enzymes, their substrates and inhibitors in 2017 and a comparison with peptidases in the PANTHER database. Nucleic Acids Res. 46, D624–D632. doi: 10.1093/nar/gkx1134, PMID: 29145643 PMC5753285

[B71] Rineau F. Stas J. Nguyen N. H. Kuyper T. W. Carleer R. Vangronsveld J.. (2016). Ectomycorrhizal fungal protein degradation ability predicted by soil organic nitrogen availability. Appl. Environ. Microbiol. 82, 1391–1400. doi: 10.1128/AEM.03191-15, PMID: 26682855 PMC4771325

[B72] Shah F. Rineau F. Canbäck B. Johansson T. Tunlid A. (2013). The molecular components of the extracellular protein-degradation pathways of the ectomycorrhizal fungus *Paxillus involutus* . New Phytol. 200, 875–887. doi: 10.1111/nph.12425, PMID: 23902518 PMC4282482

[B73] Smith S. E. Read D. J. (2010). Mycorrhizal Symbiosis (London, United Kingdom, Academic Press).

[B74] Stajich E. Tsai C. (2023). PHYling for Phylogenomic reconstruction from genomes (Version 2.0) (GitHub). Available online at: https://github.com/stajichlab/phyling (Accessed October 2024).

[B75] Steenwyk J. L. Li Y. Shen X.-X. Rokas A. (2020). ClipKIT: A multiple sequence alignment trimming software for accurate phylogenomic inference. PloS Biol. 18, e3001007. doi: 10.1371/journal.pbio.3001007, PMID: 33264284 PMC7735675

[B76] Takaya N. (2002). Dissimilatory nitrate reduction metabolisms and their control in fungi. J. bioscience bioengineering 94, 506–510. doi: 10.1016/S1389-1723(02)80187-6, PMID: 16233342

[B77] Tamm C. O. (1991). Nitrogen in terrestrial ecosystems: Questions of productivity, vegetational changes, and ecosystem stability (Berlin, Germany: Springer).

[B78] Tang P. Ning C. Liu T. Tan Z. Wang X. Liu S.. (2024). Impact of nitrogen enrichment on the functions of extracellular enzymes in ectomycorrhizal community and nutrient status of pine seedlings. Appl. Soil ecology: section Agriculture Ecosyst. Environ. 202, 105576.

[B79] Theron L. W. Divol B. (2014). Microbial aspartic proteases: current and potential applications in industry. Appl. Microbiol. Biotechnol. 98, 8853–8868. doi: 10.1007/s00253-014-6035-6, PMID: 25269600

[B80] Vaaje-Kolstad G. Tuveng T. R. Mekasha S. Eijsink V. G. H. (2019). “Enzymes for modification of chitin and chitosan,” in Chitin and Chitosan (John Wiley & Sons, Ltd, Chichester, UK), 189–228.

[B81] van der Linde S. Suz L. M. Orme C. D. L. Cox F. Andreae H. Asi E.. (2018). Environment and host as large-scale controls of ectomycorrhizal fungi. Nature 558, 243–248. doi: 10.1038/s41586-018-0189-9, PMID: 29875410

[B82] Veneault-Fourrey C. Commun C. Kohler A. Morin E. Balestrini R. Plett J.. (2014). Genomic and transcriptomic analysis of Laccaria bicolor CAZome reveals insights into polysaccharides remodelling during symbiosis establishment. Fungal Genet. biology: FG B 72, 168–181. doi: 10.1016/j.fgb.2014.08.007, PMID: 25173823

[B83] Viborg A. H. Terrapon N. Lombard V. Michel G. Czjzek M. Henrissat B.. (2019). A subfamily roadmap of the evolutionarily diverse glycoside hydrolase family 16 (GH16). J. Biol. Chem. 294, 15973–15986. doi: 10.1074/jbc.RA119.010619, PMID: 31501245 PMC6827312

[B84] Vincent F. Yates D. Garman E. Davies G. J. Brannigan J. A. (2004). The three-dimensional structure of the N-acetylglucosamine-6-phosphate deacetylase, NagA, from *Bacillus subtilis*: a member of the urease superfamily. J. Biol. Chem. 279, 2809–2816. doi: 10.1074/jbc.M310165200, PMID: 14557261

[B85] Wang J. Han S. Wang C. Li M.-H. (2022). Long-term nitrogen-addition-induced shifts in the ectomycorrhizal fungal community are associated with changes in fine root traits and soil properties in a mixed Pinus koraiensis forest. Eur. J. Soil Biol. 112, 103431. doi: 10.1016/j.ejsobi.2022.103431

[B86] Wang T. Persson P. Tunlid A. (2021). A widespread mechanism in ectomycorrhizal fungi to access nitrogen from mineral-associated proteins. Environ. Microbiol. 23, 5837–5849. doi: 10.1111/1462-2920.15539, PMID: 33891367

[B87] Wang T. Tian Z. Tunlid A. Persson P. (2020). Nitrogen acquisition from mineral-associated proteins by an ectomycorrhizal fungus. New Phytol. 228, 697–711. doi: 10.1111/nph.16596, PMID: 32279319

[B88] Waterhouse A. M. Procter J. B. Martin D. M. A. Clamp M. Barton G. J. (2009). Jalview Version 2–a multiple sequence alignment editor and analysis workbench. Bioinf. (Oxford England) 25, 1189–1191. doi: 10.1093/bioinformatics/btp033, PMID: 19151095 PMC2672624

[B89] Williamson G. Bizior A. Harris T. Pritchard L. Hoskisson P. A. Javelle A. (2024). Biological ammonium transporters from the Amt/Mep/Rh superfamily: mechanism, energetics, and technical limitations. Bioscience Rep. 44, BSR20211209. doi: 10.1042/BSR20211209, PMID: 38131184 PMC10794816

[B90] Willmann A. Weiss M. Nehls U. (2007). Ectomycorrhiza-mediated repression of the high-affinity ammonium importer gene AmAMT2 in *Amanita muscaria* . Curr. Genet. 51, 71–78. doi: 10.1007/s00294-006-0106-x, PMID: 17072660

[B91] Yang Y. Sossah F. L. Li Z. Hyde K. D. Li D. Xiao S.. (2020). Genome-wide identification and analysis of chitinase GH18 gene family in *Mycogone perniciosa* . Front. Microbiol. 11, 596719. doi: 10.3389/fmicb.2020.596719, PMID: 33505368 PMC7829358

[B92] Zeng H. Wang Z. Wang J. Yu Y. Luo W. Li H.. (2025). Genomic analysis of Pseudomonas sp. GWSMS-1 isolated from Antarctica reveals its potential in Chitin hydrolysis. BMC genomic Data 26, 43. doi: 10.1186/s12863-025-01335-0, PMID: 40615846 PMC12228359

