# Supplementary figures and images for: Diverse nitrogen acquisition strategies of conifer-associated ectomycorrhizal fungi shape unique responses to changing nitrogen regimes
Source: Front Plant Sci. 2025 Sep 23;16:1666003. doi: 10.3389/fpls.2025.1666003 (PMC12501645; doi:10.3389/fpls.2025.1666003)

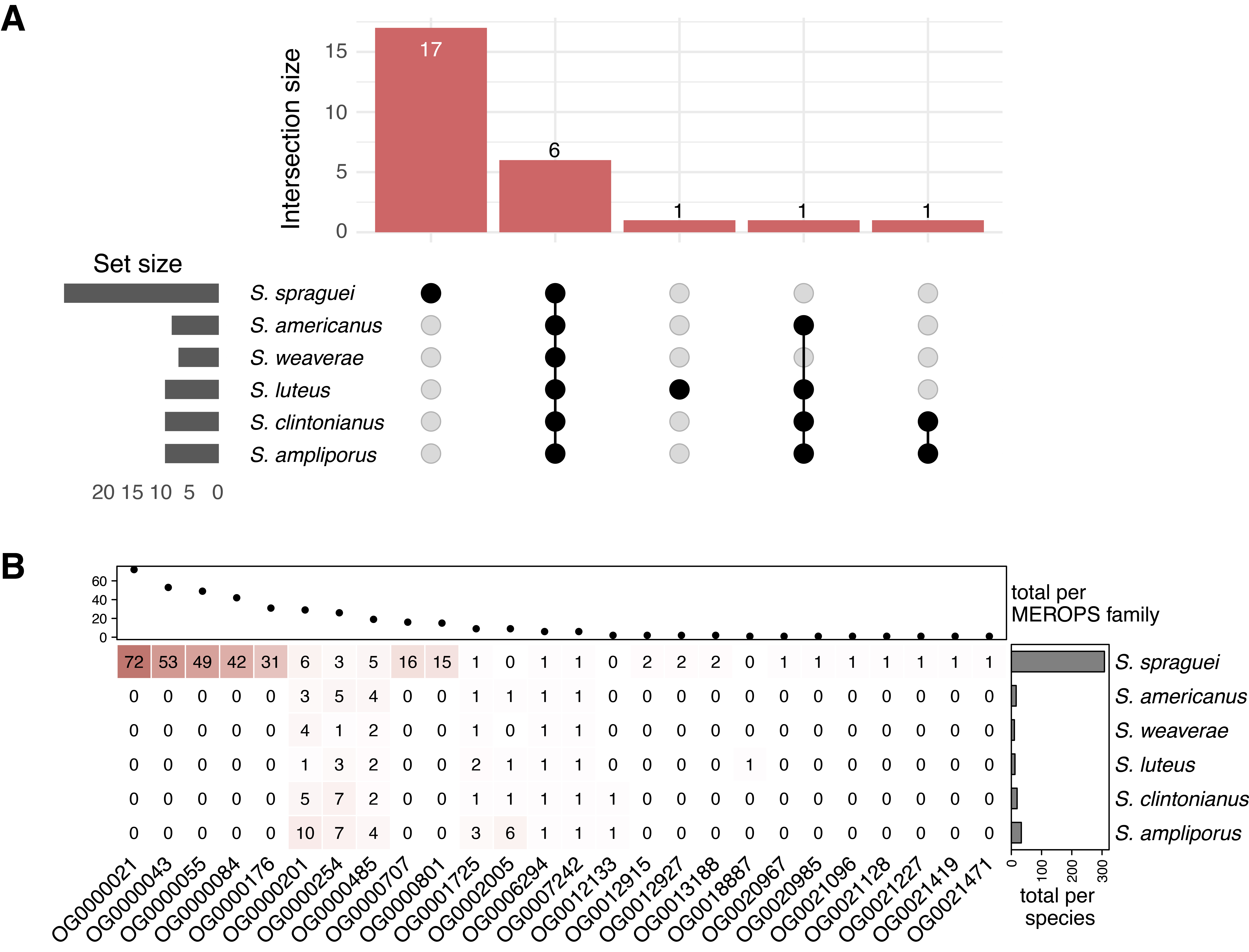

Supplement: Supplementary Figure 1 — Distribution and conservation of polyporopepsins. (A) Presence/absence conservation of orthogroups encoding polyporopepsins across Suillus species. (B) Copy number variation within individual polyporopepsin orthogroups, highlighting multiple gene amplifications in S. spraguei. [file Image1.jpeg]

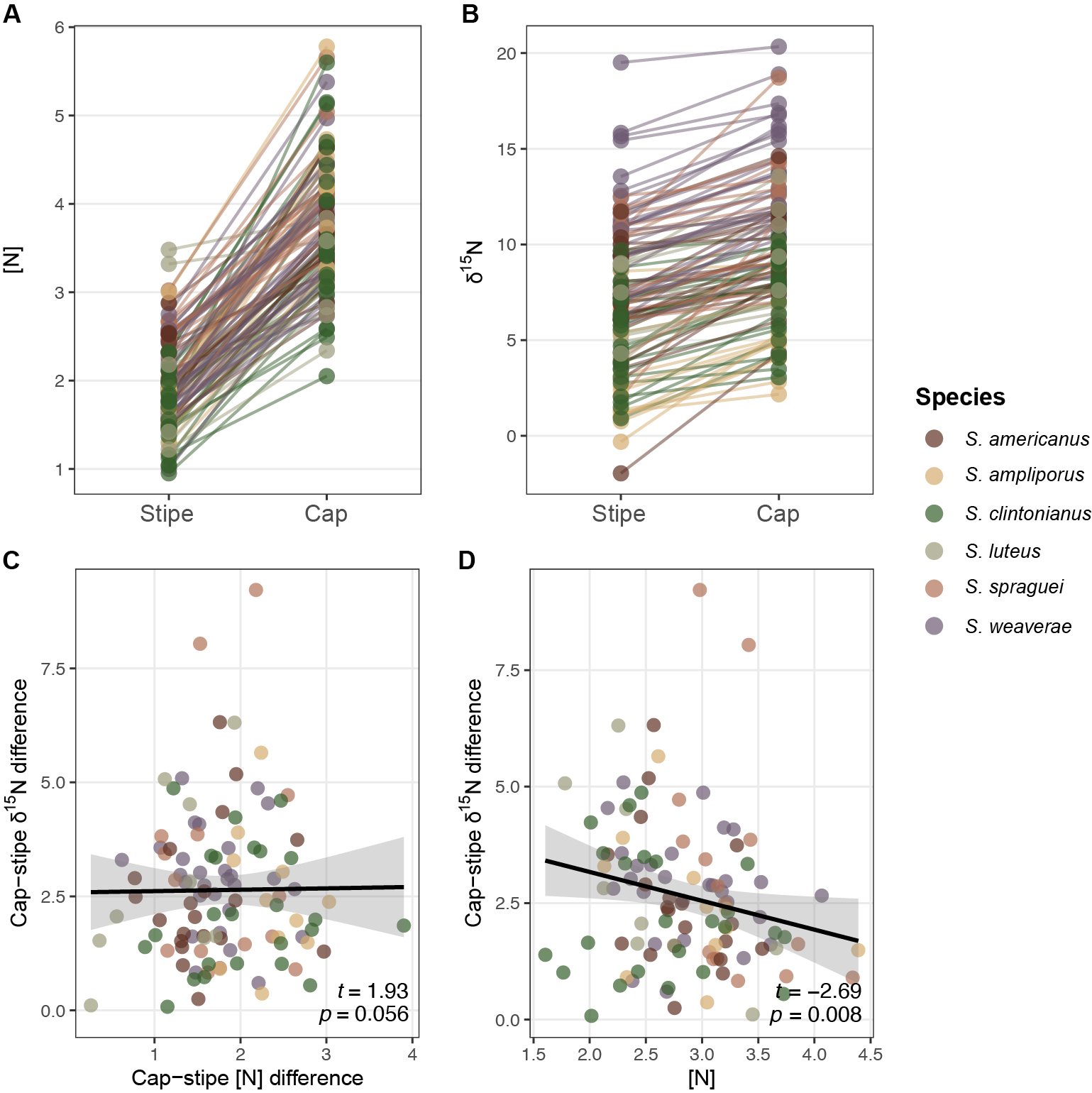

Supplement: Supplementary Figure 2 — Difference between cap and stipe δ15N. (N) concentration A) and δ15N concentration (B) were higher in cap tissue than stipe tissue. The difference between cap and stipe δ15N (C), was negatively related to average sporocarp N concentration and marginally positively related to the difference between cap and stipe N concentration (D). All graphs are color coded by species. Lines in (A, B) connecting stipe-cap datapoints represent samples taken from the same fruitbody. In (C, D) shading indicates the 95% confidence interval, and solid black lines representing the overall linear trend. [file Image2.jpeg]
